# Optical polarization perturbed by shear strains of ultrasonic bulk waves in anisotropic semiconductors: Multiphysics modeling and optoacoustic validation

**DOI:** 10.1016/j.pacs.2023.100540

**Published:** 2023-08-05

**Authors:** Yi He, Hoon Sohn, Osamu Matsuda, Zhongqing Su

**Affiliations:** a Department of Mechanical Engineering, The Hong Kong Polytechnic University, Kowloon, Hong Kong Special Administrative Region; b Department of Civil and Environmental Engineering, Korea Advanced Institute of Science and Technology, Daejeon 34141, Republic of Korea; c Center for 3D Printing Nondestructive Testing, Korea Advanced Institute of Science and Technology, Daejeon 34141, Republic of Korea; d Division of Applied Physics, Faculty of Engineering, Hokkaido University, Sapporo 060-8628, Japan

**Keywords:** Optoacoustic characterization, Photoelasticity, Anisotropic monocrystalline semiconductor, Optical polarization, Multiphysics modeling

## Abstract

Characterization of lattice properties of monocrystalline semiconductors (MS) has been rapidly advanced. Of particular interest is the use of shear strains induced by optoacoustic-bulk-waves. However, this technique has been hindered owing to the lack of quantitative correlations between optoacoustic-bulk-waves-induced shear strains and anisotropic photoelasticity of MS. Motivated by this, a multiphysics model is developed to interrogate the coupling phenomena and interaction between optical polarization and shear strains in MS. With the model, perturbation to the polarization of a monochromatic laser beam, upon interacting with optoacoustic waves in MS, is scrutinized quantitatively. Experimental results are in agreement with those from the model, both revealing the polarization perturbed by shear strains quantitatively depends on the crystal orientation and crystal-structure-related symmetry, which are jointly governed by mechanical/photoelastic/optical anisotropies of MS. The approach has paved a new way for selectively acquiring high-sensitivity shear components of optoacoustic-ultrasonic-waves for *in situ*, high-definition characterization of anisotropic MS.

## 1 Introduction

The past half a century has witnessed burgeoning semiconductors progressing from the micro-scale in dimensions to the nano-scale [1]. Recent breakthroughs in quantum research [2], high-precision manufacturing [3], and photolithography [4] have made it possible to carve three-dimensional (3-D), nanoscopic architectures inside tiny chips [5], [6], [7], and accommodate demanding needs from the third generation of semiconductors [8]. The miniaturized semiconductors have shined in a broad spectrum of applications, including, but to name a few, photoelectric sensors [9], microchips [10] and microelectromechanical systems [11]*.* In these applications, the transistor [12], packaging solder bumps [13], or through-package-vias [14] in semiconductors are of multiscale features from micrometer through nanometer.

Along with the progressive downsizing of semiconductors yet with the increased complexity in their interiors, high-precision material characterization and defect inspection of semiconductors from the onset of manufacturing phase becomes crucial to warrant a high degree of reliability and durability of the fabricated semiconductors [15]. In this connection, the X-ray micro-tomography [16], infrared thermography [17], scanning electron microscope [18], and scanning acoustic microscopy [19] have proven their effectiveness in accessing semiconductor interiors of different scales.

Nevertheless, prevailing techniques for microscopic inspection and characterization of semiconductors, in particular the monocrystalline semiconductors (MS), are often restricted by the natures of off-line operation, near-surface-only sensitivity, intrusion to inspected objects due to the use of water-based couplant or irradiation of high-intensity electron beam, time-consuming processing, and contact-type measurement (direct contact or via couplant). To circumvent some of these limitations, optoacoustic (OA) characterization-based inspection has been increasingly preferred [20], [21], [22], [23], [24], [25], [26]. Most approaches in this category employ a pulsed laser beam, referred to as the *pump beam*, to irradiate the surface of an object under inspection and to induce thermoelastically ultrasonic waves; another laser beam, referred to as the *probe beam*, in the meantime illuminates the object surface for acquiring the pump beam-induced thermoelastic ultrasonic waves propagating in the object. Theories of generating OA waves have been well established [23], [27], [28], [29], [30], [31], [32], [33], [34]. In particular, the theory based on the classical coupling effect between the thermodynamics and elasticity [35], [36], rigorously describes the mechanism of optoacoustic wave generation in objects of macroscopic scales. When OA characterization-based inspection is extended to objects having microscopic or even smaller scales, the two-temperature model [28], [30], [32], [37] has been proven effect of interpreting the ultrafast (of the order of sub-picosecond) generation of OA waves which are known as hypersound [37] or GHz phonon [30], [38]. Two-temperature model reveals phonons are generated via the sequential energy transportation from photons, electrons, to phonons, spanning a broad acoustic spectrum ranging from 10 GHz to 1 THz [39], [40]. Being a multiphysical process, the OA method brings various factors dominating the generation of GHz phonons. Most representatively, Christian, Guray, and Humphrey et al. [33], [34] theoretically and experimentally resealed the generation process of GHz bulk phonons, discovering the spatial form of phonons depends on thermal conduction, diffusivity, and electron diffusion. With the understanding of GHz phonons, Humphrey et al. [41] further commercialized their approach as the automated instrument (Onto Innovation®) for use in computer chip fabrication.

In addition to the generation of ultrasonic waves, OA method has also been used for acquisition of ultrasonic waves [24] via refractometry [42], laser Doppler vibrometry [43], interferometry [44], reflectometry [45], *etc*. Liu and Yi et al. [22], [46] estimated thickness of the coating layer on a moving silicon wafer, using a femtosecond-laser-induced ultrasound and the iris-based beam distortion detection. Liu et al. [47] measured ultrafast nonlinear ultrasonic waves and quantified the extracted wave nonlinearity, for identifying nanoscale cracks and microscale grain structures in silicon wafers. Antoncecchi et al. [48] presented an OA microscope, to image buried structures and opaque grating structures in silica substrates, with micrometer-scale transverse resolution and nanometer-scale depth sensitivity – an accuracy that is comparable to that of the scanning electron microscope or atomic force microscope.

Prevailing OA methods use the longitudinal mode of the OA ultrasonic bulk waves (OA-UBWs), as it is naturally the sole wave mode of OA-UBW generated in an elastically isotropic medium by a pump beam with a uniformly distributed irradiance [49]. When propagating in MS, the anisotropy of the monocrystalline properties of MS results in multiple wave modes that are co-existent in the principal coordinate system (P-CSYS), including shear wave mode. Indeed, the shear strain induced by the shear wave mode carries essential information of the transverse lattice features of MS, which can be important supplement to the longitudinal wave mode-induced compressional strain. Because the behavior of compressional strain only reflects the material features along the principal axes of the anisotropic properties of the medium, while that of shear strain is related to material characteristics transverse to the principal axes.

This has motivated recent endeavors to explore and make use of the shear strains induced by OA-UBW in crystalline materials, with paradigms including nondestructive evaluation of microscopic elastic properties of monocrystalline silicon [50], calibration of the crystalline grain orientation of transparent ceria ceramic substrates [51], and sound-velocity based work function analysis for semiconducting two-dimensional electronic materials [52]. Success of these applications has further inspired the use of shear strains for characterization of MS. Gusev [53] theoretically demonstrated that the plane shear waves can be generated in a submicron layer made of an elastically isotropic materials, by means of a laser-induced transient grating, to quantify the shear rigidity and shear viscosity of the layer. Matsuda et al. [54], [55] revealed that the shear strains of OA-UBW are separated at an isotropic-anisotropic interface as a result of the mode conversion when a pump beam irradiates an elastically anisotropic material with a broken symmetry of elasticity. Taking a step further, the same authors [56], [57] discovered that the shear strains of OA waves can be generated in a medium with a metallic diffraction grating. To optically capture the shear strains induced by OA waves in monocrystalline materials, specific photoelastic materials that are optically transparent (*e.g.*, silica) are coated on the sample surface under the oblique irradiation of a probe beam [49], [54], [55], [57], [58], [59]. On the basis of the significant photoelasticity in silica, the reflectance varies with regard to shear strains, which is then used to record the signal of OA-UBW.

However, when extended to the inspection of MS, multiple factors restrict the utilization of shear strains of OA-UBW. The crystal orientations of commonly used MS-wafers, *e.g.*, 〈100〉-, 〈111〉-, and 〈110〉-oriented monocrystalline silicon wafers, do not break the symmetry of the elasticity of MS. As a result, for regular MS-wafers considered in this study, shear strains and compressional strains simultaneously exist in OA-UBW, causing difficulty isolating shear strains from captured OA-UAW signals. In the meantime, lithography of the metallic diffraction grating and silica plating on MS-wafers are challenging, owing to the lack of manufacturing techniques that are available to achieve mass production and reluctance of introducing extra silica layers. Restricted by these, effort to directly measure the shear strains of OA-UBW in MS-wafer remains a daunting task, irrespective of some recent progresses in using the shear strains to evaluate the anisotropic photoelasticity of silicon [60], [61].

In recognition of the above-mentioned challenges when the shear strains induced by OA-UBW are attempted to characterize MS, a new OA approach is developed in this study. The proposed OA approach is expected to provide selective acquisition of the shear strains and facilitate *in situ* characterization of the microscale anisotropic properties of monocrystalline silicon wafers in electronics industry. In this proof-of-concept investigation, the laser pulse in the nanosecond range is employed, and the shear strains of ultrasonic bulk waves with MHz-range frequencies are measured. To start with, a multiphysics model is established to describe the mutual interaction between the optical polarization and shear strains in anisotropic MS of diamond cubic crystal structures. With the model, perturbation to the optical polarization, when a monochromatic laser beam interacts with OA-UBW propagating in the MS, is quantified. Experiment is conducted, in which the optical polarization perturbated by OA-UBW in Zinc-coated monocrystalline silicon wafers of different crystal orientations is measured, to verify the proposed approach.

## 2 OA-UBW in MS: generation and propagation

**Fig. 1:**
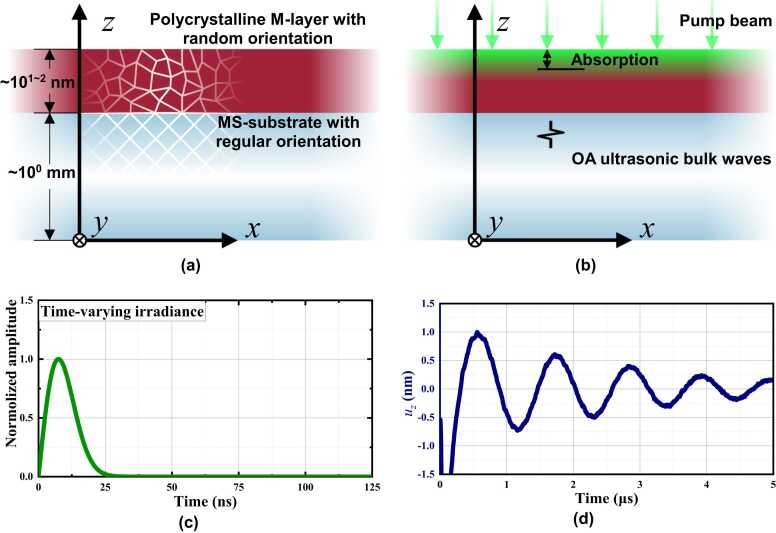
Schematic of the generation and propagation of OA-UBW in a MS wafer: (a) 2-D view of the MS wafer cross-section; (b) propagation of ultrasonic bulk waves in the substrate triggered by OA; (c) time-varying irradiance of the pulsed pump beam; (d) numerical solutions to $u_{z}$.

Consider a typical MS wafer, which is a multilayered, stacked structure consisting of a substrate of MS (MS-substrate) of a millimeter-scale thickness, and a metallic coating layer (M-layer) of a thickness of a few ten or hundred nanometers, as illustrated in Fig. 1 (a). During manufacturing, it is a common practice to insert a chromium film of a thickness of a few nanometers between the MS-substrate and the M-layer for bonding enhancement. Given its much thinner thickness compared with those of the MS-substrate and the metallic coating layer, the chromium film can be reasonably neglected. M-layer is fabricated by magnetron-sputtering of a specific metallic substance (*e.g.*, gold, platinum or zinc) on the MS-substrate, making M-layer mechanically and optically polycrystalline and isotropic. The MS-substrate, a cut from a block of MS, remains the original mechanical and optical natures of MS.

To induce ultrasonic bulk waves in the MS wafer using OA excitation, a pulsed pump beam irradiates the M-layer normally, with the principle illustrated schematically in Fig. 1 (b). The M-layer absorbs partial energy of the pump beam, and its volume is expanded simultaneously. Consider that the dimension of the illuminated area of the pump beam is six-order of magnitude larger than the nanoscale thickness of the M-layer, the OA-UBW in the isotropic M-layer is a longitudinal plane wave propagating downwards only (see Fig. 1 (b)) – it means that the *z*-axis component of the particulate displacement is non-zero, while those in the other two directions are zero.

Upon penetrating the surface of the M-layer, the pump beam propagates perpendicularly in the M-layer as an irradiance attenuating exponentially. Denoting the time-varying irradiance of the pump beam as $I_{pump}\left(t\right)$ (here t is the time), the relationship between the increment of temperature along *z*-axis ΔT(z,t) (here, z is the *z*-axis coordinate) and $I_{pump}\left(t\right)$ can be governed, in terms of the heat equation, as [44].

(1) $$\frac{\partial \Delta T\left(z,t\right)}{\partial t}=\frac{a_{m}}{\rho_{m}C_{m}}\frac{\partial^{2}\Delta T\left(z,t\right)}{\partial z^{2}}+I_{pump}\left(t\right)\frac{1-r_{m}}{\rho_{m}C_{m}h_{m,abs}}e^{\frac{z-h_{wafer}}{h_{m,abs}}},$$

where $r_{m}$ signifies the reflectivity of the M-layer that is associated with the optical wavelength of the pump beam $\lambda_{pump}$. $a_{m}$, $\rho_{m}$ and $C_{m}$ represent the thermal conductivity, density, and specific heat capacity of the M-layer, respectively. e is the base of the natural logarithm. $h_{wafer}$ signifies the thickness of the MS-wafer (including both the M-layer and MS-substrate). $h_{m,abs}$ denotes the absorption depth of the M-layer associated with $\lambda_{pump}$. For a pulsed pump beam, $I_{pump}\left(t\right)$ is formulated as

(2) $$I_{pump}\left(t\right)=A_{pump}\sqrt{e}\left(\omega_{pump}t\right)e^{-\frac{{\left(\omega_{pump}t\right)}^{2}}{2}},\;\mathrm{when}\;t\geq 0,$$

where $A_{pump}$ and $\omega_{pump}$ are the peak amplitude and the central angular frequency of the time-varying irradiance of $I_{pump}\left(t\right)$, respectively. Fig. 1 (c) presents the result calculated via Eq. (2). Substituting Eq. (2) into (1), ΔT(z,t) and further particulate displacements in OA-UBW can be obtained. As mentioned earlier, because only the longitudinal mode of OA-UBW exists in the M-layer, the *z*-components of the stress ($\sigma_{m,zz}$) and strain ($\xi_{m,zz}$) in the M-layer, in the form of tensor, satisfy the following equation [23]:

(3) $$\sigma_{m,zz}=\frac{E_{m}\left(1-v_{m}\right)}{\left(1+v_{m}\right)\left(1-2v_{m}\right)}\xi_{m,zz}+\sigma_{m-T,zz},\xi_{m,zz}=\frac{\partial u_{z}}{\partial z},$$

where $E_{m}$ and $v_{m}$ signify the elastic modulus and Poisson’s ratio of the M-layer, respectively. $u_{z}$ is the *z*-component of particulate displacement. $\sigma_{m-T,zz}$ is the *z*-component of the thermal compressional stress tensor that is caused by variation in temperature ΔT(z,t), as

(4) $$\sigma_{m-T,zz}=-\frac{E_{m}}{\left(1-2v_{m}\right)}\alpha_{m}\Delta T\left(z,t\right),$$

where $\alpha_{m}$ is the coefficient of thermal expansion of the M-layer.

With (3), (4), the OA-UBW, expressed in terms of obtained $\sigma_{m,zz}$ and $u_{z}$, can thus be defined as

(5) $$\frac{\partial \sigma_{m,zz}}{\partial z}=\rho_{m}\frac{\partial^{2}u_{z}}{\partial t^{2}}.$$

Substituting (3), (4) into (5), Eq. (5) can be rephrased as

(6) $$c_{l-m}^{2}\frac{\partial^{2}u_{z}}{\partial z^{2}}-\frac{E_{m}\alpha_{m}}{\left(1-2v_{m}\right)\rho_{m}}\frac{\partial \Delta T\left(z,t\right)}{\partial z}=\frac{\partial^{2}u_{z}}{\partial t^{2}},\;c_{l-m}^{2}=\frac{E_{m}\left(1-v_{m}\right)}{\left(1+v_{m}\right)\left(1-2v_{m}\right)\rho_{m}}.$$

In Eq. (6), $c_{l-m}$ represents the phase velocity of the longitudinal wave propagating in the M-layer. The numerical solution to $u_{z}$, corresponding to the time-varying irradiance in Eq. (2), can be obtained using a finite difference method upon applying the constrain of boundary and initial conditions of $\sigma_{m,zz}=0$ (where $z=h_{wafer}$), $\xi_{m,zz}=0$ and $\frac{\partial \xi_{m,zz}}{\partial t}=0$ (both when t=0).

**Fig. 2:**
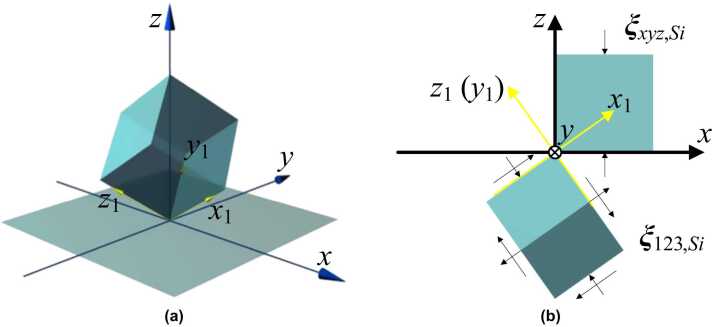
(a) Definition of G-CSYS and P-CSYS of the MS-substrate, in which *x*- *y*- and *z*-axes belong to G-CSYS, and $x_{1}$-, $y_{1}$- and $z_{1}$-axes are in P-CSYS; (b) the lateral view of two coordinate systems along the positive direction of the *y*-axis, showing the components of strain tensors in G-CSYS and P-CSYS (under a plane strain condition).

When the generated OA-UBW arrives at the interface between the MS-substrate and M-layer (where $z=h_{wafer}-h_{M-layer}$; $h_{M-layer}$ is the thickness of the M-layer and therefore $h_{wafer}-h_{M-layer}$ signifies the thickness of the MS-substrate), part of its energy is reflected due to both the particulate displacement continuity along the *z*-axis and the mismatched material properties. Letting the MS-substrate be silicon with symmetric orientations along *z*-axis, and $u_{z,Si}$ be the particulate displacement of OA-UBW in the MS-substrate, the ratio between the amplitudes of $u_{z}$ and $u_{z,Si}$, denoted with $r_{zz,m-Si}$, is

(7) $$r_{zz,m-Si}=\frac{2\rho_{m}c_{l-m}}{\rho_{m}c_{l-m}+\rho_{Si}c_{l,z-Si}},$$

where $\rho_{Si}$ is the density of the MS-substrate. On the other hand, it is worthy of attention that Eq. (7) requires further generalization if the interested orientation along the *z*-axis is not symmetric [51]. Additionally, $c_{l,z-Si}$ is the phase velocity of the longitudinal mode in the MS-substrate along the *z*-axis, which is given by

(8) $$c_{l,z-Si}=\sqrt{\frac{{\bar{C}}_{zz,Si}}{\rho_{Si}}},$$

where ${\bar{C}}_{zz,Si}$ represents the element in the third row and third column of the silicon stiffness matrix with respect to the global coordinate system (G-CSYS), as shown in Fig. 2 (a), ${\bar{C}}_{Si}$, to be ascertained by linearly transforming the material stiffness matrix from P-CSYS to the G-CSYS, $C_{Si}$, using rotation matrices for stress and strain tensors, $T_{\sigma }$ and $T_{\xi }$, via

(9) $${\overset{¯}{C}}_{Si}=T_{\sigma }^{-1}C_{Si}T_{\xi },$$

where

(10) $$\begin{array}{c} C_{Si}=\left[\begin{array}{cccccc} C_{11,Si} & C_{12,Si} & C_{12,Si} & 0 & 0 & 0\\ C_{12,Si} & C_{11,Si} & C_{12,Si} & 0 & 0 & 0\\ C_{12,Si} & C_{12,Si} & C_{11,Si} & 0 & 0 & 0\\ 0 & 0 & 0 & C_{44,Si} & 0 & 0\\ 0 & 0 & 0 & 0 & C_{44,Si} & 0\\ 0 & 0 & 0 & 0 & 0 & C_{44,Si} \end{array}\right]\\ T_{\sigma }=\left[\begin{array}{cccccc} \chi_{x_{1}}^{2} & \beta_{x_{1}}^{2} & \gamma_{x_{1}}^{2} & 2\chi_{x_{1}}\beta_{x_{1}} & 2\chi_{x_{1}}\gamma_{x_{1}} & 2\beta_{x_{1}}\gamma_{x_{1}}\\ \chi_{y_{1}}^{2} & \beta_{y_{1}}^{2} & \gamma_{y_{1}}^{2} & 2\chi_{y_{1}}\beta_{y_{1}} & 2\chi_{y_{1}}\gamma_{y_{1}} & 2\beta_{y_{1}}\gamma_{y_{1}}\\ \chi_{z_{1}}^{2} & \beta_{z_{1}}^{2} & \gamma_{z_{1}}^{2} & 2\chi_{z_{1}}\beta_{z_{1}} & 2\chi_{z_{1}}\gamma_{z_{1}} & 2\beta_{z_{1}}\gamma_{z_{1}}\\ \chi_{x_{1}}\chi_{y_{1}} & \beta_{x_{1}}\beta_{y_{1}} & \gamma_{x_{1}}\gamma_{y_{1}} & \chi_{x_{1}}\beta_{y_{1}}+\chi_{y_{1}}\beta_{x_{1}} & \chi_{x_{1}}\gamma_{y_{1}}+\chi_{y_{1}}\gamma_{x_{1}} & \beta_{x_{1}}\gamma_{y_{1}}+\beta_{y_{1}}\gamma_{x_{1}}\\ \chi_{x_{1}}\chi_{z_{1}} & \beta_{x_{1}}\beta_{z_{1}} & \gamma_{x_{1}}\gamma_{z_{1}} & \chi_{x_{1}}\beta_{z_{1}}+\chi_{z_{1}}\beta_{x_{1}} & \chi_{x_{1}}\gamma_{z_{1}}+\chi_{z_{1}}\gamma_{x_{1}} & \beta_{x_{1}}\gamma_{z_{1}}+\beta_{z_{1}}\gamma_{x_{1}}\\ \chi_{y_{1}}\chi_{z_{1}} & \beta_{y_{1}}\beta_{z_{1}} & \gamma_{y_{1}}\gamma_{z_{1}} & \chi_{y_{1}}\beta_{z_{1}}+\chi_{z_{1}}\beta_{y_{1}} & \chi_{y_{1}}\gamma_{z_{1}}+\chi_{z_{1}}\gamma_{y_{1}} & \beta_{y_{1}}\gamma_{z_{1}}+\beta_{z_{1}}\gamma_{y_{1}} \end{array}\right],\\ T_{\xi }=\left[\begin{array}{cccccc} \chi_{x_{1}}^{2} & \beta_{x_{1}}^{2} & \gamma_{x_{1}}^{2} & \chi_{x_{1}}\beta_{x_{1}} & \chi_{x_{1}}\gamma_{x_{1}} & \beta_{x_{1}}\gamma_{x_{1}}\\ \chi_{y_{1}}^{2} & \beta_{y_{1}}^{2} & \gamma_{y_{1}}^{2} & \chi_{y_{1}}\beta_{y_{1}} & \chi_{y_{1}}\gamma_{y_{1}} & \beta_{y_{1}}\gamma_{y_{1}}\\ \chi_{z_{1}}^{2} & \beta_{z_{1}}^{2} & \gamma_{z_{1}}^{2} & \chi_{z_{1}}\beta_{z_{1}} & \chi_{z_{1}}\gamma_{z_{1}} & \beta_{z_{1}}\gamma_{z_{1}}\\ 2\chi_{x_{1}}\chi_{y_{1}} & 2\beta_{x_{1}}\beta_{y_{1}} & 2\gamma_{x_{1}}\gamma_{y_{1}} & \chi_{x_{1}}\beta_{y_{1}}+\chi_{y_{1}}\beta_{x_{1}} & \chi_{x_{1}}\gamma_{y_{1}}+\chi_{y_{1}}\gamma_{x_{1}} & \beta_{x_{1}}\gamma_{y_{1}}+\beta_{y_{1}}\gamma_{x_{1}}\\ 2\chi_{x_{1}}\chi_{z_{1}} & 2\beta_{x_{1}}\beta_{z_{1}} & 2\gamma_{x_{1}}\gamma_{z_{1}} & \chi_{x_{1}}\beta_{z_{1}}+\chi_{z_{1}}\beta_{x_{1}} & \chi_{x_{1}}\gamma_{z_{1}}+\chi_{z_{1}}\gamma_{x_{1}} & \beta_{x_{1}}\gamma_{z_{1}}+\beta_{z_{1}}\gamma_{x_{1}}\\ 2\chi_{y_{1}}\chi_{z_{1}} & 2\beta_{y_{1}}\beta_{z_{1}} & 2\gamma_{y_{1}}\gamma_{z_{1}} & \chi_{y_{1}}\beta_{z_{1}}+\chi_{z_{1}}\beta_{y_{1}} & \chi_{y_{1}}\gamma_{z_{1}}+\chi_{z_{1}}\gamma_{y_{1}} & \beta_{y_{1}}\gamma_{z_{1}}+\beta_{z_{1}}\gamma_{y_{1}} \end{array}\right]. \end{array}$$

In Eq. (10), $C_{11,Si}$, $C_{12,Si}$ and $C_{44,Si}$ are the three independent stiffness coefficients. $\chi_{i_{P}}$, $\beta_{i_{P}}$ and $\gamma_{i_{P}}$ ($i_{P}=x_{1},y_{1},z_{1}$) are the direction cosines of the $i_{P}$-axis in P-CSYS with regard to the *x*-, *y*-, and *z*-axes in G-CSYS, respectively. On the other hand, using Eq. (7), $u_{z,Si}$ can be expressed as (for z>0 and $<h_{wafer}-h_{M-layer}$)

(11) $$u_{z,Si}=r_{zz,m-Si}\cdot u_{z}\left(\frac{c_{l-m}}{c_{l,z-Si}}z,t\right).$$

It is noted that the independent variable *z* in either $u_{z}$ or $u_{z,Si}$ has an implicit coefficient equal to the wavenumber of OA-UBW propagating in the corresponding medium. The ratio between two wavenumbers in the MS-substrate and in the M-layer is equal to $c_{l-m}/c_{z,l-Si}$. Consequently, *z* is modulated in Eq. (11) by multiplying this ratio. The numerical solution to Eq. (11) is shown in Fig. 1 (d). Eq. (11) states that only the particulate displacement along the *z*-axis exists in the MS-substrate, which is a consequence of the displacement continuity at the interface between the M-layer and the MS-substrate perpendicular to the *z*-axis. Given an arbitrary crystal orientation of the MS wafer, in Fig. 2 (b), $T_{\xi }$ can be used to convert the strain tensor of the MS-substrate in G-CSYS, $\xi_{xyz,Si}$, to its counterpart in P-CSYS, $\xi_{123,Si}$, as

(12) $$\xi_{123,Si}=T_{\xi }\xi_{xyz,Si}=T_{\xi }{\left[\begin{array}{cccccc} 0 & 0 & \frac{\partial u_{z-Si}}{\partial z} & 0 & 0 & 0 \end{array}\right]}^{T}.$$

In the above, superscript “*T*” represents the transpose operation of a matrix or vector. From (10), (12) is noted that at least two of *i*_*p*_-axis-, and one of the shear components of $\xi_{123,Si}$ co-exist in the MS-substrate.

With Eq. (12), all the components of the strain tensors of OA-UBW can be obtained in P-CYCS (in contrast, some components of the strain tensors do not exist in G-CYCS including the shear strains). In what follows, $\xi_{123,Si}$ as defined in Eq. (12) is to be recalled, in conjunction with the photoelasticity of MS-substrate, to quantify perturbation to the optical polarization by OA-UBW.

## 3 Multiphysics modeling of perturbation to optical polarization by OA-UBW

**Fig. 3:**
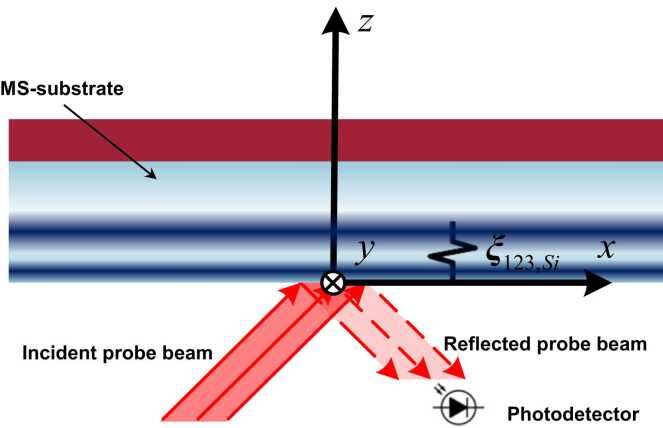
Schematic of the monochromatic probe beam interacting with OA-UBW at the lower surface of the sample.

Subsequent to the generation of OA-UBW in the MS-substrate from the sample surface (*i.e.*, upper surface in Fig. 3, a monochromatic probe beam obliquely illuminates another surface (lower surface in the figure) of the MS wafer. In view of the anisotropic photoelasticity of the sample, the permittivity of the MS-substrate oscillates along with the propagation of OA-UBW. And thus, after the probe beam is reflected from the surface, its electromagnetic field varies that can be captured by a photodetector. In this study, the variation of the electromagnetic field signifies the perturbation to the polarization of the reflected probe beam. The strain induced by OA-UBW is naturally inhomogeneous along the *z*-axis. To approximate the influence of *z*-inhomogeneity of OA-UBW-induced strains, a strategy of segmenting the strains into multiple thin strained layers in sequence is considered here, each of which is thin enough to be safely considered as homogeneous. After the segmentation, a multiplicative chain rule is adopted to calculate the collective effect of all thin strained layers on the polarization of the reflected probe beam.

### 3.1 Shear strain-induced perturbation to permittivity

The OA-UBW-induced strain in the MS-substrate shifts the permittivity of silicon, and correlates the electric displacement field with the electric field of the probe beam. As shown in Eq. (12), obtained from the *z*-inhomogeneous $\xi_{xyz,Si}$, $\xi_{123,Si}$ also varies along the *z*-axis. As a result, a direct analysis using $\xi_{123,Si}$ may incur difficulty obtaining an explicit solution. To circumvent this, first assume that the MS-substrate contains a homogeneous strain in P-CSYS, referred as to $\xi_{123,Si-homo}$. Owing to the anisotropic photoelasticity of silicon, $\xi_{123,Si-homo}$ varies the relative dielectric impermeability tensor of the MS-substrate in P-CSYS, $\Delta \kappa_{123,Si,r}$, as

(13) $$\Delta \kappa_{123,Si,r}=P_{123,Si}\xi_{123,Si-homo},$$

where

(14) $$P_{123,Si}=\left[\begin{array}{cccccc} p_{11,Si} & p_{12,Si} & p_{12,Si} & 0 & 0 & 0\\ p_{12,Si} & p_{11,Si} & p_{12,Si} & 0 & 0 & 0\\ p_{12,Si} & p_{12,Si} & p_{11,Si} & 0 & 0 & 0\\ 0 & 0 & 0 & p_{44,Si} & 0 & 0\\ 0 & 0 & 0 & 0 & p_{44,Si} & 0\\ 0 & 0 & 0 & 0 & 0 & p_{44,Si} \end{array}\right].$$

In the above, $P_{123,Si}$ is the photoelasticity matrix of the MS-substrate in P-CSYS ($p_{11,Si}$, $p_{12,Si}$ and $p_{44,Si}$ being the three independent photoelastic coefficients of $P_{123,Si}$). For the convenience of discussion, an operator, ${\left(\cdot \right)}_{matrix}$, which converses a tensor in the form of a vector, is introduced here. With it, converting $\Delta \kappa_{123,Si,r}$ into its matrix-form yields

(15) $${\left(\Delta \kappa_{123,Si,r}\right)}_{matrix}=\left[\begin{array}{ccc} \Delta \kappa_{1,Si,r} & \Delta \kappa_{6,Si,r} & \Delta \kappa_{5,Si,r}\\ \Delta \kappa_{6,Si,r} & \Delta \kappa_{2,Si,r} & \Delta \kappa_{4,Si,r}\\ \Delta \kappa_{5,Si,r} & \Delta \kappa_{4,Si,r} & \Delta \kappa_{3,Si,r} \end{array}\right],$$

where $\Delta \kappa_{k_{0},Si,r}$ ($k_{0}=1,\ldots ,6$) is the $k_{0}$-th element of $\Delta \kappa_{123,Si,r}$. With the mutually inverse relationship between the matrix-form of the relative permittivity and the matrix-form of the relative inverse permittivity tensors, the matrix-form of the perturbed relative inverse permittivity tensor of the MS-substrate in P-CSYS, ${\left(\kappa_{123,p-Si,r}\right)}_{matrix}$, can be obtained via

(16) $$\begin{array}{c} {\left(\kappa_{123,p-Si,r}\right)}_{matrix}={\left(\varepsilon_{123,Si,r}\right)}_{matrix}^{-1}+{\left(\Delta \kappa_{123,Si,r}\right)}_{matrix},\\ {\left(\varepsilon_{123,Si,r}\right)}_{matrix}=\left[\begin{array}{ccc} \varepsilon_{Si,r} & 0 & 0\\ 0 & \varepsilon_{Si,r} & 0\\ 0 & 0 & \varepsilon_{Si,r} \end{array}\right]\;. \end{array}$$

In the above equation, $\varepsilon_{123,Si,r}$ is the relative permittivity tensor of the unstrained MS-substrate in P-CSYS, and $\varepsilon_{Si,r}$ is the relative permittivity of the unstrained MS-substrate associated with the wavelength of the probe beam. Noting the mathematic commonality between the stress tensor and $\kappa_{123,p-Si,r}$, as implied in Eq. (13), which resembles Hooke's law, the perturbed permittivity tensor of the MS-substrate in G-CSYS can be calculated via

(17) $${\left(\varepsilon_{xyz,p-Si}\right)}_{matrix}=\varepsilon_{0}{\left(T_{\sigma }^{-1}\kappa_{123,p-Si,r}\right)}_{matrix}^{-1},$$

where $\varepsilon_{0}$ denotes the permittivity of vacuum. Eq. (17) characterizes the anisotropic permittivity of the MS-substrate in terms of $\xi_{123,Si-homo}$. The OA-UBW-induced-shear strains will induce a new electric field in the MS-substrate which is orthogonal to the original electric field of the incident probe beam. The interaction between the new and original electric fields theoretically introduces the fourth to the sixth components of $\varepsilon_{xyz,p-Si}$ in Eq. (17) (*i.e.*, the off-diagonal components of ${\left(\varepsilon_{xyz,p-Si}\right)}_{matrix}$). In another word, the absence of shear strains in the MS substrate will not induce any off-diagonal component of ${\left(\varepsilon_{xyz,p-Si}\right)}_{matrix}$. This conclusion from the above derivation has implied that the presence or absence of the off-diagonal components of ${\left(\varepsilon_{xyz,p-Si}\right)}_{matrix}$ can be associated with existence or inexistence of shear strains in the MS-substrate, respectively.

### 3.2 Probe beam in homogeneously strained MS-substrate

**Fig. 4:**
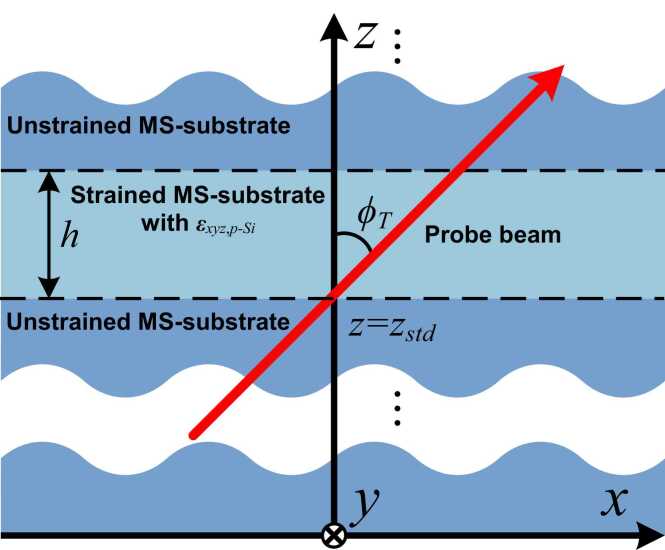
Schematic of the oblique propagation of the probe beam in the MS-substrate with $\varepsilon_{xyz,p-Si}$, in which the icon “⊗” indicates the positive direction of *y*-axis faces the *x*-*z* plane inward.

Upon obtaining $\varepsilon_{xyz,p-Si}$ with $\xi_{123,Si-homo}$, the oblique propagation of the probe beam in the MS-substrate with a homogenously strained layer is to be discussed, namely the scenario as sketched in Fig. 4. Let $\phi_{T}$ be the angle between the propagation direction of the probe beam and the positive direction of the z-axis, $z_{std}$ be the *z*-coordinate of the lower boundary between the homogenously stained layer and unstrained MS-substrate, and *h* be the thickness of the homogenously strained layer. Specific to this study, the perturbation to optical polarization due to the homogenously strained layer is concerned, which is related to the electromagnetic fields of the probe beam where *z* = *z*_*std*_ and *z* = *z*_*std*_+*h*. Therefore, given an incident probe beam with a specific electromagnetic field, the solution to the electromagnetic field of the probe beam transmitted out, where *z* = *h*, is of particular interest.

Let $E_{T}$ be the electric field of the probe beam. defined as

(18) $$E_{T}=E_{x,T}x_{0}+E_{y,T}y_{0}+E_{z,T}z_{0},$$

where, $x_{0}$, $y_{0}$, and $z_{0}$ are the unit vectors along the *x*, *y*, and *z*-axes, respectively. Here, the probe beam propagates in the *xz*-plane – the plane of laser incidence, which indicates that $E_{T}$ is *y*-independent (*i.e.*, without *y*-component in the optical wavevector). To this end, the components of $E_{T}$ in Eq. (18) can be expanded as

(19) $$\begin{array}{c} E_{x,T}=A_{x,E,T}e^{j(\omega_{probe}t-k_{x,T}x-k_{z,T}z)},\\ E_{y,T}=A_{y,E,T}e^{j(\omega_{probe}t-k_{x,T}x-k_{z,T}z)},\\ E_{z,T}=A_{z,E,T}e^{j(\omega_{probe}t-k_{x,T}x-k_{z,T}z)}, \end{array}$$

where $E_{i,T}$ and $A_{i,E,T}$ (i=x,y,z) are the *i*-components of $E_{T}$ and the amplitude of $E_{i,T}$, respectively. $\omega_{probe}$, $k_{x,T}$ and $k_{z,T}$ denote the circular frequency, the *x*-, and *z*-components of the optical wavevector of the probe beam, respectively. j is the imaginary unit. Governed by the Maxwell’s equations, the relationship between $E_{T}$ and electric displacement $D_{T}$, and the relationship between the magnetic field $H_{T}$ and magnetic flux density $B_{T}$, can be generalized as

(20) $$\left[\begin{array}{cccccc} 0 & 0 & 0 & 0 & -\partial /\partial z & \partial /\partial y\\ 0 & 0 & 0 & \partial /\partial z & 0 & -\partial /\partial x\\ 0 & 0 & 0 & -\partial /\partial y & \partial /\partial x & 0\\ 0 & \partial /\partial z & -\partial /\partial y & 0 & 0 & 0\\ -\partial /\partial z & 0 & \partial /\partial x & 0 & 0 & 0\\ \partial /\partial y & -\partial /\partial x & 0 & 0 & 0 & 0 \end{array}\right]\left[\begin{array}{c} E_{x,T}\\ E_{y,T}\\ E_{z,T}\\ H_{x,T}\\ H_{y,T}\\ H_{z,T} \end{array}\right]=j\omega_{probe}\left[\begin{array}{c} D_{x,T}\\ D_{y,T}\\ D_{z,T}\\ B_{x,T}\\ B_{y,T}\\ B_{z,T} \end{array}\right],$$

where $D_{i,T}$, $H_{i,T}$ and $B_{i,T}$ are the *i*-components of $D_{T}$, $H_{T}$ and $B_{T}$, respectively. With the *y*-independence, the item of ∂/∂y retreats to zero. Consequently, Eq. (20) can be simplified as

(21) $$\left[\begin{array}{cccccc} 0 & 0 & 0 & 0 & jk_{z,T} & 0\\ 0 & 0 & 0 & -jk_{z,T} & 0 & jk_{x,T}\\ 0 & 0 & 0 & 0 & -jk_{x,T} & 0\\ 0 & -jk_{z,T} & 0 & 0 & 0 & 0\\ jk_{z,T} & 0 & -jk_{x,T} & 0 & 0 & 0\\ 0 & jk_{x,T} & 0 & 0 & 0 & 0 \end{array}\right]\left[\begin{array}{c} E_{x,T}\\ E_{y,T}\\ E_{z,T}\\ H_{x,T}\\ H_{y,T}\\ H_{z,T} \end{array}\right]=j\omega_{probe}\left[\begin{array}{c} D_{x,T}\\ D_{y,T}\\ D_{z,T}\\ B_{x,T}\\ B_{y,T}\\ B_{z,T} \end{array}\right].$$

On the other hand, $D_{T}$ and $B_{T}$ can be defined, by assuming that silicon has no intrinsic capability of optical rotation, as

(22) $$\left[\begin{array}{cc} {\left(\varepsilon_{xyz,p-Si}\right)}_{matrix} & 0_{3\times 3}\\ 0_{3\times 3} & {\left(\mu_{xyz,Si}\right)}_{matrix} \end{array}\right]\left[\begin{array}{c} E_{x,T}\\ E_{y,T}\\ E_{z,T}\\ H_{x,T}\\ H_{y,T}\\ H_{z,T} \end{array}\right]=\left[\begin{array}{c} D_{x,T}\\ D_{y,T}\\ D_{z,T}\\ B_{x,T}\\ B_{y,T}\\ B_{z,T} \end{array}\right],$$

where $0_{3\times 3}$ is the null matrix with a dimension of 3 × 3. $\mu_{xyz,Si}$ is the permeability tensor of the MS-substrate, which reads

(23) $${\left(\mu_{xyz,Si}\right)}_{matrix}=\left[\begin{array}{ccc} \mu_{Si} & 0 & 0\\ 0 & \mu_{Si} & 0\\ 0 & 0 & \mu_{Si} \end{array}\right],$$

where $\mu_{Si}$ is the permeability of the MS-substrate. Substituting Eq. (22) into Eq. (21) yields

(24) $$\left[\begin{array}{c} k_{z,T}H_{y,T}\\ -k_{z,T}H_{x,T}+k_{x,T}H_{z,T}\\ -k_{x,T}H_{y,T}\\ -k_{z,T}E_{y,T}\\ k_{z,T}E_{x,T}-k_{x,T}E_{z,T}\\ k_{x,T}E_{y,T} \end{array}\right]=\omega_{probe}\left[\begin{array}{c} \varepsilon_{1,xyz,p-Si}E_{x,T}+\varepsilon_{6,xyz,p-Si}E_{y,T}+\varepsilon_{5,xyz,p-Si}E_{z,T}\\ \varepsilon_{6,xyz,p-Si}E_{x,T}+\varepsilon_{2,xyz,p-Si}E_{y,T}+\varepsilon_{4,xyz,p-Si}E_{z,T}\\ \varepsilon_{5,xyz,p-Si}E_{x,T}+\varepsilon_{4,xyz,p-Si}E_{y,T}+\varepsilon_{3,xyz,p-Si}E_{z,T}\\ \mu_{Si}H_{x,T}\\ \mu_{Si}H_{y,T}\\ \mu_{Si}H_{z,T} \end{array}\right].$$

In Eq. (24), $\varepsilon_{k_{0},xyz,p-Si}$ is the $k_{0}$-th element of $\varepsilon_{xyz,p-Si}$. With the second and fifth algebraic equations in Eq. (24), $H_{z,T}$ and $E_{z,T}$ can be represented in terms of $E_{x,T}$, $E_{y,T}$, $H_{x,T}$ and $H_{y,T}$. With that, Eq. (24) is rephrased as

(25) $$\left(\omega_{probe}\Delta -k_{z,T}I_{4\times 4}\right)\psi_{T}=0_{4\times 1},$$

where

(26) $$\begin{array}{c} \psi_{T}={\left[\begin{array}{cccc} E_{x,T} & H_{y,T} & E_{y,T} & -H_{x,T} \end{array}\right]}^{T},\\ \Delta =\left[\begin{array}{cccc} -\frac{\varepsilon_{5,xyz,p-Si}k_{x,T}}{\varepsilon_{3,xyz,p-Si}\omega_{probe}} & \mu_{Si}-\frac{k_{x,T}^{2}}{\omega_{probe}^{2}\varepsilon_{3,xyz,p-Si}} & -\frac{\varepsilon_{4,xyz,p-Si}k_{x,T}}{\varepsilon_{3,xyz,p-Si}\omega_{probe}} & 0\\ \varepsilon_{1,xyz,p-Si}+\frac{\varepsilon_{5,xyz,p-Si}k_{z,T}}{k_{x,T}} & -\frac{\varepsilon_{5,xyz,p-Si}\omega_{probe}\mu_{Si}}{k_{x,T}} & \varepsilon_{6,xyz,p-Si} & 0\\ 0 & 0 & 0 & \mu_{Si}\\ \varepsilon_{6,xyz,p-Si}+\frac{\varepsilon_{4,xyz,p-Si}k_{z,T}}{k_{x,T}} & -\frac{\varepsilon_{4,xyz,p-Si}\omega_{probe}\mu_{Si}}{k_{x,T}} & \varepsilon_{2,xyz,p-Si}-\frac{k_{x,T}^{2}}{\omega_{probe}^{2}\mu_{Si}} & 0 \end{array}\right]. \end{array}$$

In the above, $\psi_{T}$ and Δ are referred as to the electromagnetic field vector of the obliquely incident probe beam, and the differential propagation matrix, respectively. $0_{4\times 1}$ is the null column vector with a dimension of 4 rows × 1 column, and $I_{4\times 4}$ is the identical matrix with a dimension of 4 rows × 4 columns. By eliminating the terms of exponential functions of $E_{i,T}$ and $H_{i,T}$, Eq. (25) is further simplified as

(27) $$\left(\omega_{probe}\Delta -k_{z,T}I_{4\times 4}\right){\;\left[\begin{array}{cccc} A_{x,E,T} & A_{y,H,T} & A_{y,E,T} & -A_{x,H,T} \end{array}\right]}^{T}=0_{4\times 1}.$$

In the above, $A_{x,H,T}$ and $A_{y,H,T}$ are the amplitudes of $H_{x,T}$ and $H_{y,T}$, respectively. To warrant a nontrivial solution to ${\left[\begin{array}{cccc} A_{x,E,T} & A_{y,H,T} & A_{y,E,T} & -A_{x,H,T} \end{array}\right]}^{T}$, Eq. (27) has to satisfy

(28) $$\det\left(\omega_{probe}\Delta -k_{z,T}I_{4\times 4}\right)=0,$$

where the operator det(⋅) signifies the matrix determinant. Recalling that Δ is associated with both $k_{x,T}$ and $k_{z,T}$, and meanwhile noting the propagating-direction constraint that $k_{x,T}=k_{z,T}\;\tan\;\phi_{T}$, Eq. (28) is therefore a quartic polynomial equation subject to $k_{z,T}$. By solving $k_{z,T}$ and substituting the obtained solutions to Eq. (27), a set of four complex solutions, ${\left[\begin{array}{cccc} A_{x,E,T} & A_{y,H,T} & A_{y,E,T} & -A_{x,H,T} \end{array}\right]}^{T}$, are obtained.

By solving Eq. (28), four solutions to $k_{z,T}$, signified as $k_{l_{0},z,T}$ ($l_{0}=1,\ldots ,4$), are obtained, based on which a simplified relation between the electromagnetic field vectors of the probe beam propagating into and outward the homogenously strained layer in the MS substrate, ${\psi_{T}|}_{z=z_{std}}$ and ${\psi_{T}|}_{z=z_{std}+h}$, as seen in Fig. 4, is obtained as

(29) $${\psi_{T}|}_{z=z_{std}+h}=L\left(h\right){\psi_{T}|}_{z=z_{std}}=A_{EM}\left[\begin{array}{cccc} e^{-jk_{1,z,T}h} & 0 & 0 & 0\\ 0 & e^{-jk_{2,z,T}h} & 0 & 0\\ 0 & 0 & e^{-jk_{3,z,T}h} & 0\\ 0 & 0 & 0 & e^{-jk_{4,z,T}h} \end{array}\right]A_{EM}^{-1}{\psi_{T}|}_{z=z_{std}},$$

In Eq. (29), $A_{EM}$ is a matrix, of which the *l*_0_-th column is the *l*_0_-th solution to ${\left[\begin{array}{cccc} A_{x,E,T} & A_{y,H,T} & A_{y,E,T} & -A_{x,H,T} \end{array}\right]}^{T}$. L(h) represents the layer matrix of the MS-substrate with $\varepsilon_{xyz,p-Si}$ and a thickness of *h*.

(28), (29) can be used not only for determining the $\phi_{T}$-off optical wavevector of the probe beam corresponding to $\omega_{probe}$, but also describing an optically anisotropic constitutive relationship between the input and output of the electric field in the probe beam, both of which are essential to the following derivation. By applying the boundary conditions where *z* = *z*_*std*_ (see Fig. 4) and *z* = *z*_*std*_+*h* (*viz.*, two interfaces between the homogenously strained layer and the unstrained MS-substrate), the electromagnetic field vectors of the reflection and transmission of the probe beam in the surfaces are obtained quantitatively, in the following session.

### 3.3 Perturbation to the polarization of probe beam

**Fig. 5:**
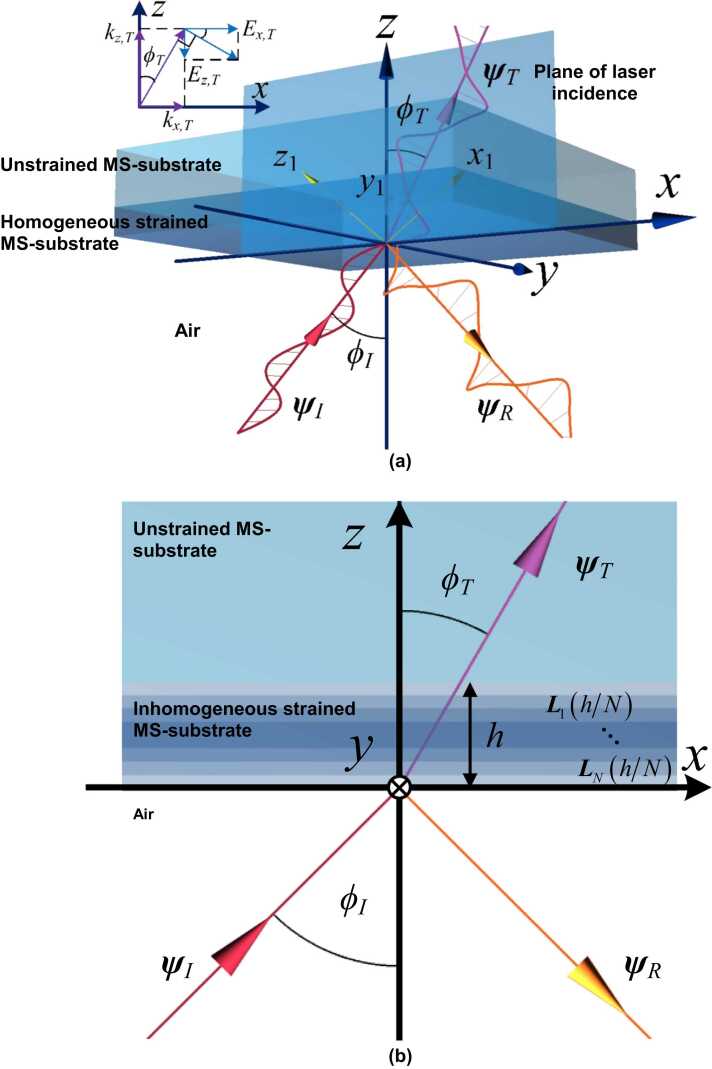
(a) Schematic of the three-layer medium, including the ambient air layer, strained and unstrained MS-substrates ($k_{x,T}$-$k_{z,T}$- and $E_{x,T}$-$E_{z,T}$-relationships are displayed in the upper-left sub-figure); (b) the approximation of the inhomogeneous strained MS-substrate, dividing it in to *N* sub-layers, each of which can be treated as a homogeneous sub-layer.

The above multiphysics modeling defines the propagation characteristics of the probe beam in the homogeneously strained MS-substrate only. In practice, the probe beam propagates through the ambient air before illuminating the sample, and then being reflected and transmitted at the boundary between the ambient air and the strained MS-substrate. To include the air coupling into the multiphysics modeling, a three-layer medium is modelized, as shown in Fig. 5 (a). In the medium, the homogeneously strained MS-substrate with a thickness of *h* is sandwiched by a semi-infinite air layer and a semi-infinite unstrained MS-substrate. The incident probe beam is initiated from the air, and obliquely illuminates the sample surface, where *z* = 0 (*z*_*std*_=0 in this case), with an incident angle of $\phi_{I}$, which is then partially reflected to the air and transmitted into the homogeneously strained MS-substrate. The transmitted laser branch continues its propagation in the homogeneously strained MS-substrate, and then in the unstrained MS-substrate.

Recalling (18), (19), (20), (21), (22), (23), (24), (25), (26), (27), (28), the derivation is also applicable to the semi-infinite air layer and the semi-infinite unstrained MS-substrate by using their respective permittivity tensors. Akin to the form of $\psi_{T}$ in Eq. (25), the electromagnetic field vectors of the incident and reflected probe beams, $\psi_{I}$ and $\psi_{R}$ respectively, are defined, respectively, as

(30) $$\begin{array}{c} \psi_{I}={\left[\begin{array}{cccc} E_{x,I} & H_{y,I} & E_{y,I} & -H_{x,I} \end{array}\right]}^{T},\\ \psi_{R}={\left[\begin{array}{cccc} E_{x,R} & H_{y,R} & E_{y,R} & -H_{x,R} \end{array}\right]}^{T}, \end{array}$$

where $E_{i,I}$ and $H_{i,I}$ denote the *i*-components (i=x,y,z) of the electric and magnetic fields of the incident probe beam, respectively; $E_{i,R}$ and $H_{i,R}$ are the counterparts of the reflected beam. Recalling Eq. (29), when *z* = 0 in $\psi_{I}$ and $\psi_{R}$ and simultaneously *z* = *h* in $\psi_{T}$, Eq. (29) retreats to

(31) $${\psi_{T}|}_{z=h}=L\left(h\right)\left({\psi_{I}|}_{z=0}+{\psi_{R}|}_{z=0}\right).$$

In the left side of Eq. (31), noting $k_{x,T}=k_{z,T}\;\tan\;\phi_{T}$ and $E_{z,T}=-E_{x,T}\;\tan\;\phi_{T}$ (seeing the upper-left subfigure in Fig. 5 (a)), and using the fourth and fifth rows of Eq. (24), $H_{x,T}$ and $H_{y,T}$ can be formulated as

(32) $$H_{x,T}=\frac{-k_{z,T}E_{y,T}}{\omega_{probe}\mu_{Si}},\mathrm{and}{\;H}_{y,T}=\frac{k_{z,T}E_{x,T}{\text{sec}}^{2}\phi_{T}}{\omega_{probe}\mu_{Si}}.$$

In the right end of Eq. (31), the incident probe beam is governed by an updated version of Eq. (28) with the permittivity and permeability of air, $\varepsilon_{air}$ and $\mu_{air}$, as:

(33) $$\det\left(\omega_{probe}\left[\begin{array}{cccc} 0 & \mu_{air}-\frac{{\left(k_{z,air}\;\tan\;\phi_{I}\right)}^{2}}{\omega_{probe}^{2}\varepsilon_{air}} & 0 & 0\\ \varepsilon_{air} & 0 & 0 & 0\\ 0 & 0 & 0 & \mu_{air}\\ 0 & 0 & \varepsilon_{air}-\frac{{\left(k_{z,air}\;\tan\;\phi_{I}\right)}^{2}}{\omega_{probe}^{2}\mu_{air}} & 0 \end{array}\right]-k_{z,air}I_{4\times 4}\right)=0.$$

With Eq. (33), the *z*-component of optical wavevector of the incident probe beam in the air, $k_{z,air}$, is obtained. It is noted that governed by the law of reflection, the reflected probe beam possesses the identical *x*-component of the optical wavevector as that of the incident probe beam, but opposite in the *z*-component. Upon obtaining $k_{z,air}$ and replacing $\mu_{Si}$ with $\mu_{air}$ in the fourth and fifth rows of Eq. (24), $H_{x,I}$, $H_{y,I}$, $H_{x,R}$ and $H_{y,R}$ can be formulated in terms of $E_{y,I}$, $E_{x,I}$, $E_{y,R}$ and $E_{x,R}$, as

(34) $$\begin{array}{ccc} H_{x,I} & = & \frac{-k_{z,air}E_{y,I}}{\omega_{probe}\mu_{air}},H_{y,I}=\frac{k_{z,air}E_{x,I}+k_{z,air}E_{x,I}\tan^{2}\phi_{I}}{\omega_{probe}\mu_{air}},\\ H_{x,R} & = & \frac{k_{z,air}E_{y,R}}{\omega_{probe}\mu_{air}},H_{y,R}=\frac{-k_{z,air}E_{x,R}+k_{z,air}E_{x,R}\tan^{2}\phi_{I}}{\omega_{probe}\mu_{air}}. \end{array}$$

In above, the signs of $H_{x,R}$ and $H_{y,R}$ either being plus or minus are determined by the orientations of $E_{y,R}$ and $E_{x,R}$, both of which are chosen as being positive along *y*- and *x*-axes, respectively. Substituting (32), (34) into (31) yields

(35) $${\left[\begin{array}{c} E_{x,T}\\ \frac{k_{z,T}E_{x,T}{\text{sec}}^{2}\phi_{T}}{\mu_{Si}}\\ E_{y,T}\\ \frac{k_{z,T}E_{y,T}}{\mu_{Si}} \end{array}\right]|}_{z=h}=L\left(h\right){\left[\begin{array}{c} \left(E_{x,I}+E_{x,R}\right)\\ \frac{k_{z,air}\left(E_{x,I}{\text{sec}}^{2}\phi_{I}-E_{x,R}\left(1-\tan^{2}\phi_{I}\right)\right)}{\mu_{air}}\\ \left(E_{y,I}+E_{y,R}\right)\\ \frac{k_{z,air}\left(E_{y,I}-E_{y,R}\right)}{\mu_{air}} \end{array}\right]|}_{z=0},$$

where $\tan\;\phi_{I}/\tan\;\phi_{T}=k_{z,T}/k_{z,air}$ (*viz.*, Snell's law). In Eq. (35), there are four separate linear algebraic equations for unknown ${E_{x,T}|}_{z=h}$, ${E_{y,T}|}_{z=h}$, ${E_{x,R}|}_{z=0}$ and ${E_{y,R}|}_{z=0}$, which are then solved with given $E_{x,I}$, $E_{y,I}$ and $\phi_{I}$ of the incident probe beam.

Up to Eq. (35), it is assumed that the strained layer in the three-layer medium is homogeneous in material properties, namely using $\xi_{123,Si-homo}$ from the beginning of Section 3. It is noteworthy that the strain tensor induced by the OA-UBW in the MS-substrate within P-CSYS, $\xi_{123,Si}$, is inhomogeneous along the *z*-axis (Eq. (12)), which results in the absence of a closed-form solution to L(h). To obtain the approximation to L(h), the inhomogeneously strained layer with $\xi_{123,Si}$ is divided into *N* sub-layers along the *z*-axis, each of which has a thickness of less than 1% of the wavelength of OA-UBW, as shown in Fig. 5 (b). Under this circumstance, each sub-layer can be deemed as homogeneous and defined using the strain tensor, $\xi_{123,Si}\left(z_{j_{0}}\right)$ ($z_{j_{0}}$ is the *z*-coordinate of $j_{0}$-th sub-layer, where $j_{0}=1,\ldots ,N$). Subsequently, the layer matrix of $j_{0}$-th sub-layer, $L_{j_{0}}\left(h/N\right)$, can be obtained upon substituting $\xi_{123,Si}\left(z_{j_{0}}\right)$ to $\xi_{123,Si-homo}$. Recalling Eq. (29), a multiplicative chain rule is expressed as

(36) $${\psi_{T}|}_{z=h}=\left(\prod_{j_{0}=1}^{N}L_{j_{0}}\left(\frac{h}{N}\right)\right)\cdot {\psi_{T}|}_{z=0}.$$

Eq. (36) indicates the approximated L(h) is the product of all $L_{j_{0}}\left(h/N\right)$. By replacing L(h) using $\prod_{j_{0}=1}^{N}L_{j_{0}}\left(h/N\right)$, Eq. (35) is also valid for the MS-substrate with $\xi_{123,Si}$. It is noteworthy that the multiplicative chain rule is applicable using the convolution, which calculates the electric field of the reflected probe beam corresponding to the differentiated local strain at first, and then convolutes the solution with the strain distribution along *z*-axis. Another alternative strategy for obtaining a solution of perturbed polarization of the probe beam is to use the Dirac function and obtain a closed-form solution corresponding to the differentiation of the strain through the Heaviside function, followed with integrating the distribution of the strain along *z*-axis.

${E_{x,T}|}_{z=h}$, ${E_{y,T}|}_{z=h}$, ${E_{x,R}|}_{z=0}$ and ${E_{y,R}|}_{z=0}$ contain all the requested information to depict polarization of the reflected and transmitted probe beams from the sample surface, respectively. As far as the reflected probe is concerned, its azimuth $\Delta \theta_{R}$ and ellipticity angle $\Delta {ο}_{R}$ are calculated via

(37) $$\Delta \theta_{R}=\frac{1}{2}\arctan\left(\frac{S_{2,R}}{S_{1,R}}\right),\Delta {ο}_{R}=\frac{1}{2}\arcsin\left(\frac{S_{3,R}}{\sqrt{S_{1,R}^{2}+S_{2,R}^{2}+S_{3,R}^{2}}}\right),$$

where

(38) S1,R=|Ey,R|z=0|2−|Ex,R|z=0cosϕI|2,S2,R=2Re(Ey,R|z=0⋅Ex,R|z=0*cosϕI),S3,R=−2Im(Ey,R|z=0⋅Ex,R|z=0*cosϕI),

where superscript “*”, operators Re(⋅) and Im(⋅) signify the conjugate, real and imaginary parts of a complex value, respectively. $S_{1,R}$, $S_{2,R}$ and $S_{3,R}$ are Stokes parameters.

The derivation as detailed from 3.1, 3.2, 3.3 theoretically exhibits the principle of the perturbation of OA-UBW-induced strain to the optical polarization of a probe beam that is reflected from a sample surface. With this and given crystal orientation of the MS wafer, the specific variation of the azimuth and ellipticity angle can be obtained analytically and quantitatively.

## 4 Experimental validation

The developed multiphysics modeling has interrogated the perturbation of OA-UBW-induced shear strains to the polarization of the probe beam reflected from the MS-substrate. To validate the effectiveness of the developed model, the experiment is conducted. In experiment, the instantaneous and temporal signals of the intensity of the reflected probe beam are acquired, and used to characterize the perturbated optical polarization.

### 4.1 Experimental set-ups

**Fig. 6:**
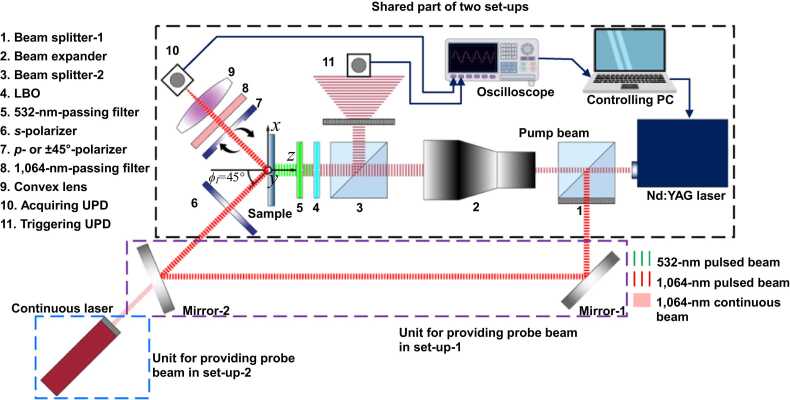
Schematic of two set-ups for observing the instantaneous perturbation to the polarization (set-up-1) and for acquiring temporal intensity signals of the polarization-perturbed reflected probe beam (set-up-2) (black frame: the shared part of two set-ups; purple and blue frames: units of providing probe beams in set-up-1 and −2, respectively.).

Two experimental set-ups are configured, as depicted in Fig. 6, respectively for observing the instantaneous perturbation to polarization of the reflected probe beam induced by OA-UBW-induced shear strains (set-up-1), and for acquiring the temporal intensity signals of a reflected continuous probe beam (set-up-2). The common instruments in both set-ups consist of a nanosecond pulsed-laser pump system (QUANTEL® Centurion+ 50 mJ), two ultrafast photodetectors (UPDs, ALPHALAS® UPD-35-UVIR-P), a digital oscilloscope (Keysight Infiniium® EXR 104 A), and a coaxial cage system (LUBON®) with a series of optical lens assemblies. The nanosecond pulsed-laser pump system is a compact pulsed Nd: YAG diode-pumped solid slate laser device, to periodically emit a pulsed pump beam. The pump beam has the pulse repetition frequency of 20 Hz, the optical wavelength of 1064 nm, the duration of 12 ns, the quasi-square cross-section of 3.2 mm × 3.2 mm, and the energy intensity of 20 mJ/pulse. Two UPDs, one for triggering the acquisition and the other for acquiring intensity signals of the reflected probe beam, has the bandwidth of up to 10 GHz, which has been proven adequately sensitive to a broad range of optical wavelengths spanning from 350 to 1700 nm. The digital oscilloscope registers the analog data from UPDs with the bandwidth of 1.05 GHz and the sampling rate of 16 GHz. A series of optical lenses installed in the coaxial cage system includes two beam splitters (the transmitting-to-reflecting split ratio is 10:1), a twofold beam expander, a lithium triborate crystal (LBO), a diffuser, two linear polarizers with an extinction ratio over 10^6^:1 for 1064-nm laser beam, a filter passing the 1064-nm laser and a convex lens.

The emitted pulsed laser beam is split by the beam-splitter-1 (see Fig. 6), of which the transmitting component serves as the pump beam, and the vertically reflected branch is the probe beam in the set-up-1:

1) for the pump beam, the size of its cross-section is doubled to be 6.4 mm × 6.4 mm via the beam expander, which is further split by the beam-splitter-2. The intensity signal of the vertically reflected component of the size-doubled pump beam is captured by the triggering UPD (here, to protect the triggering UPD, a diffuser is inserted between the triggering UPD and the Beam splitter-2 to reduce the irradiance). The LBO halves the optical wavelength of the transmitting component of the size-doubled pump beam to 532 nm, to prevent the pump beam from being interfered with the probe beam. After passing through the LBO, the pump beam still contains partial laser components of the wavelength of 1064 nm. To eliminate these components, a 532-nm-passing filter is used to screen out all light components except those with the wavelength of 532 nm. Subsequently, the 532-nm, size-doubled pump beam irradiates the surface of the sample and generates the OA-UBW to propagate in the MS sample.
2) for the probe beam, a linear *s*-polarizer (“*s*-” means the passing axis of a linear polarizer is perpendicular to the plane of laser incidence) is applied to *s*-polarize the probe beam. Another linear polarizer extracts the intensity signals of the reflected probe beam with the shear-strain-perturbed polarization, of which the passing axis can be shifted to be parallel with (referring as to “*p*-” hereafter) or ± 45° to the plane of incidence, this giving flexibility in measurement in which the responses of the *p*- or ± 45°-polarization of reflected probe beam can be captured. The filter passes the light of the wavelength of 1064 nm only and minimizes the effect of ambient light interference. The convex lens focuses the reflected probe beam after the beam passes two polarizers, to increase the irradiance intensity, and thus to enhance the signal-to-noise ratio of the intensity signals to be captured by the acquiring UPD.

All instruments and the sample are immobilized on an optical platform, immune to ambient vibration. The key difference between the two set-ups is the units that are used to provide probe beams. In set-up-1, the vertically reflected component of the original pulsed laser beam acts as the probe beam. The pulsed probe beam is obliquely reflected twice by the two mirrors, to irradiate the back surface of the sample with a $\phi_{I}$ of 45°. A temporal delay of 12.5 ns (that means the probe beam travels additional 3.75 m in the free space compared with the pump beam), is applied in experiment. By manipulating this delay in time, in conjunction with a time of 7.5 ns which a pulsed probe beam takes to reach its peak from excitation, the peaks of the pulsed probe beam and OA-UBW are temporally aligned, ensuring the highest sensitivity of acquiring the instantaneously perturbed polarization of the reflected probe beam. In set-up-2, the 1,064-nm probe beam with the averaged power of 30 mW is provided by a continuous laser with an identical $\phi_{I}$. The continuous illumination enables the registration of temporal intensity signals of the polarization-perturbed reflected probe beam. The back-side access of the probe beam is owing to the fact that the photoelastic acquisition requires the direct illumination on the MS-substrate. In addition, this also excludes the interference from the residual-heat noise of the pump beam on the probe beam.

### 4.2 Specimens

**Fig. 7:**
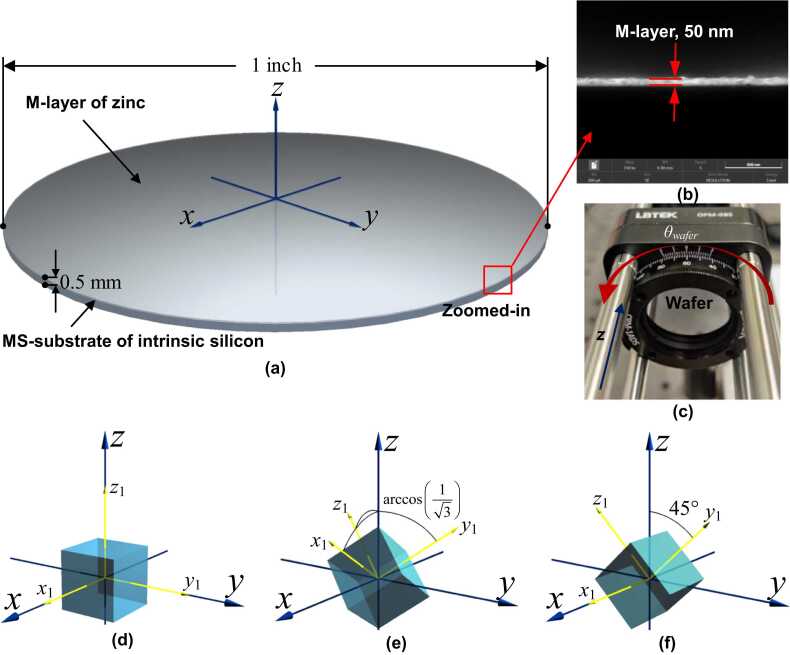
(a) The monocrystalline silicon wafer used in experiment; (b) SEM image of the wafer showing the nanoscopic stratified structure of the M-layer; (c) wafer installed in a rotatable mount with vernier scale; (d-f) P-CSYSs ($x_{1}$-, $y_{1}$-, and $z_{1}$-axes) and G-CSYSs (*x*-, *y*-, and *z*-axes) of 〈100〉-, 〈111〉-, and 〈110〉-crystal.

Industrial grade intrinsic monocrystalline silicon wafers with 〈100〉-, 〈111〉-, and 〈110〉- crystal orientations (referred to as 〈100〉-, 〈111〉-, and 〈110〉-Si-wafers, hereinafter) are acquired, each having a diameter of 1 in. (25.4 mm) and a nominal thickness of 0.5 mm, and both surfaces of wafers are polished. On one surface of each wafer, a 50 nm-thick M-layer, is deposited using the zinc substance, which has a higher coefficient of thermal expansion compared with other commonly available coating metals, Fig. 7 (a). Key material parameters of the monocrystalline silicon and the polycrystalline zinc are listed in Table 1. The nanoscale stratified structure of the wafer is exhibited in the image, Fig. 7 (b), obtained using scanning electronic microscopy (SEM). The wafer is installed in a rotatable mount with vernier scales, Fig. 7 (c), which rotates the wafer about *z*-axis with a precision of 0.2°.

**Table 1:** Key material properties of polycrystalline zinc, monocrystalline silicon, air, and vacuum [60], [61], [62], [63], [64], [65], [66], [67].

Polycrystalline zinc		Monocrystalline intrinsic silicon	
$r_{m}$	0.87 [63]	$C_{11,Si}$ (GPa)	165.60[64]
$a_{m}$ (W/(m⋅K))	110 [62]	$C_{12,Si}$ (GPa)	63.90[64]
$C_{m}$ (J/(kg⋅K))	388 [62]	$C_{44,Si}$ (GPa)	79.50[64]
$\rho_{m}$ (${\mathrm{kg}/m}^{3}$)	7140 [62]	$\rho_{Si}$ (${\mathrm{kg}/m}^{3}$)	2330.00[64]
$E_{m}$ (GPa)	108.0 [67]	$p_{11,Si}$	-9.27 × 10^−2^[60], [61]
$v_{m}$	0.25 [67]	$p_{12,Si}$	1.93 × 10^−2^[60], [61]
$\alpha_{m}$	32.5 × 10^−6^[62]	$p_{44,Si}$	7.50 × 10^−2^[60], [61]
——————	——————	$\varepsilon_{Si,r}$	12.64[65]
——————	——————	$\mu_{Si}$ (H/m)	1.25658 × 10^−6^[62]
Air		Vacuum	
$\varepsilon_{air}$ (F/m)	8.854105 × 10^−12^[62], [66]	$\varepsilon_{0}$ (F/m)	8.854188 × 10^−12^[62]
$\mu_{air}$ (H/m)	1.256638 × 10^−6^[62], [66]	$\mu_{0}$ (H/m)	1.256637 × 10^−6^[62]

The P-CSYSs (at the initial position, without rotation) and G-CSYSs of 〈100〉-, 〈111〉-, and 〈110〉-crystal orientations are presented in Fig. 7 (d)-(f), respectively. For the 〈100〉-crystal orientation, *x*_1_*-*, *y*_1_*-*, and *z*_1_*-*axes are, respectively, the *x-*, *y-*, and *z-*axes of the G-CSYS. In the 〈111〉-crystal orientation, all of the three axes in P-CSYS are $\arctan\left(1/\sqrt{3}\right)$ to *z-*axis, and *x*_1_*-*axis lies on the *x*-*z*-plane. In the 〈110〉-crystal orientation, *x*_1_*-* and *x-*axes overlap, yet *y*_1_*-*, and *z*_1_*-*axes are 45° to *z-*axis. The G-CSYS remains the same for all specimens, while P-CSYS is synchronously rotated when the wafer is rotated. The angle of rotating the wafer about *z*-axis, $\theta_{wafer}$, is defined as the anticlockwise angle between the *x*-*z*-plane and *x*_1_*-z*-plane.

### 4.3 Key experimental procedure

For observing the instantaneous perturbation of the OA-UBW-induced shear strains to polarization of the reflected probe beam, the 532-nm, size-doubled pump beam irradiates the center of the M-layer of the sample, and the acquisition is triggered using the triggering UPD, in Fig. 6. Simultaneously, the double-reflected probe beam split from the beam splitter-1 is *s*-polarized and then illuminates the center of the uncoated surface of the sample. To observe the intensity of the *p*-polarized component of the reflected probe beam, the passing axis of the second polarizer is turned to be parallel to the plane of incidence. Upon being filtered by the 1064-nm filter and focused through the convex lens, the *p*-polarized intensity signal is captured using the acquiring UPD. The above procedure is repeated at each deca-degree as the wafer rotates. In each acquisition, 2000 peak values are captured and averaged to minimize the ambient noise interference and measurement uncertainty.

**Fig. 8:**
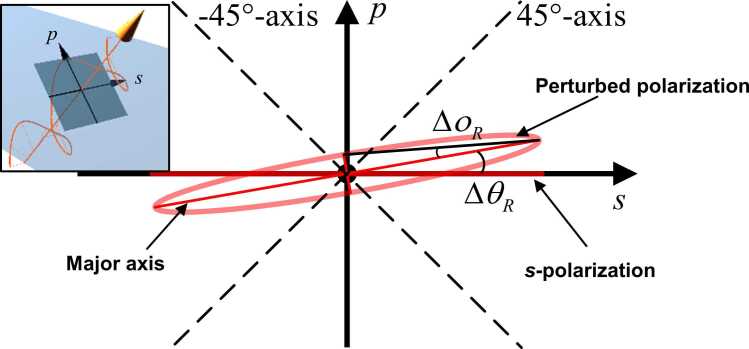
Schematic of the geometric relationship between *s*-polarized and perturbed polarization (*ps*-coordinate system lies in the cross-section of the probe beam as shown in the insert).

It is noteworthy that the *p*-polarized intensity from the *s*-polarized probe beam is related to both $\Delta \theta_{R}$ and $\Delta {ο}_{R}$ that are mutually coupled. In experimental, it is challenging to directly extract $\Delta \theta_{R}$ and $\Delta {ο}_{R}$ from the sole *p*-polarized intensity signals. In addition, identifying the phase of the electric field of the probe beam is not feasible using set-up-1, because the acquiring UPD captures the optical intensity only. This leads to the failure of obtaining $\Delta \theta_{R}$ and $\Delta {ο}_{R}$ experimentally through Eq. (37). As an alternative, ± 45°-polarized intensity signals are required (Fig. 8). Supposing the measured peak values of *p*- and ± 45°-polarized intensity signals are $P_{p}$, $P_{45^{{}^{\circ}}}$ and $P_{-45^{{}^{\circ}}}$, respectively, and considering the fact that intensity is proportional to the square of electric field, the geometric relationship among $\Delta \theta_{R}$, $\Delta {ο}_{R}$, $P_{p}$, $P_{45^{{}^{\circ}}}$ and $P_{-45^{{}^{\circ}}}$ is formulated as

(39) $$\begin{array}{c} \sqrt{P_{major}}\left(\tan\;\Delta {ο}_{R}\;\cos\;\Delta \theta_{R}+\;\sin\;\Delta \theta_{R}\right)=\sqrt{P_{p}},\\ \sqrt{P_{major}}\left(\tan\;\Delta {ο}_{R}\;\cos\left(45^{{}^{\circ}}+\Delta \theta_{R}\right)+\;\cos\left(45^{{}^{\circ}}-\Delta \theta_{R}\right)\right)=\sqrt{P_{45^{{}^{\circ}}}},\\ \sqrt{P_{major}}\left(\tan\;\Delta {ο}_{R}\;\cos\left(45^{{}^{\circ}}-\Delta \theta_{R}\right)+\;\cos\left(45^{{}^{\circ}}+\Delta \theta_{R}\right)\right)=\sqrt{P_{-45^{{}^{\circ}}}}, \end{array}$$

where $P_{major}$ is the amplitude of the intensity signal along the major axis of the polarization perturbed by OA-UBW-induced shear strains. Unknown variables $P_{major}$, $\Delta \theta_{R}$ and $\Delta {ο}_{R}$ are to be obtained by solving Eq. (39) with the measured $P_{p}$, $P_{45^{{}^{\circ}}}$ and $P_{-45^{{}^{\circ}}}$.

For obtaining the temporal intensity signals, set-up-2 is employed. A continuous, 1064-nm probe beam is emitted by the continuous laser, which passes through the same assembly of lenses as set-up-1, is *s*-polarized by the *s*-polarizer and then illuminates the center of the uncoated surface of the MS wafer. The polarization of the probe beam of set-up-2 is continuously perturbed by the shear strains of OA-UBW in the MS wafer. The digital oscilloscope continuously registers the variating *p*-polarized intensity signals, which characterize the strains of OA-UBW.

## 5 Results and discussion

The intensity signals of the reflected probe beam, associated with different crystal orientations of the MS, obtained theoretically and experimentally, are analyzed for interrogating the characteristics of the perturbation of OA-UBW-induced shear strains to polarization of the probe beam.

### 5.1 Intensity ratio between *p*- and *s*-states of polarization

**Fig. 9:**
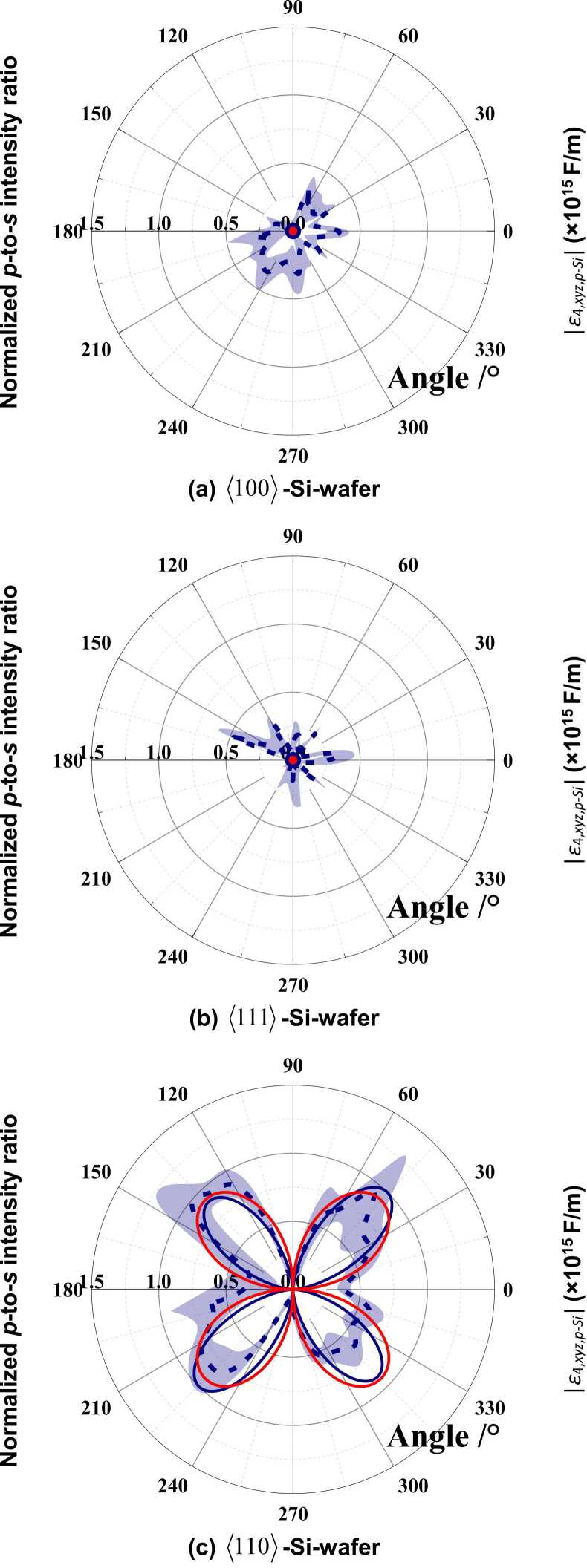
The *p*-to-*s* intensity ratios and $\left|\varepsilon_{4,xyz,p-Si}\right|$ associated with $\theta_{wafer}$: (a) 〈100〉-Si-wafer; (b) 〈111〉-Si-wafer; (c) 〈110〉-Si-wafer (red solid line: theoretical $\left|\varepsilon_{4,xyz,p-Si}\right|$; blue solid line: theoretical *p*-to-*s* intensity ratio; blue dot-shape line: experimental *p*-to-*s* intensity ratio with error bar displayed as the light blue area).

The intensity ratios between *p*- and *s*-states of polarization (*p*-to-*s* intensity ratio), corresponding to the reflected and incident probe beams, are calculated using (13), (14), (15), (16), (17), (18), (19), (20), (21), (22), (23), (24), (25), (26), (27), (28), (29), (30), (31), (32), (33), (34), (35), (36) for 〈100〉-, 〈110〉-, and 〈111〉-Si-wafers, respectively. The corresponding experimental results measured using the set-up-1 are shown in Fig. 9. All the calculated and experimental results are normalized with regard to their respective maxima. The theoretical results predict that, regardless of the existence of OA-UBW, there is no observable perturbation of the OA-UBW-induced shear strains to the polarization of the reflected probe beam in the 〈100〉- and 〈111〉-Si-wafers. Specifically, for 〈100〉-Si-wafer, the absence of the perturbation is the result of the null shear components in $\xi_{123,Si}$, regardless of $\theta_{wafer}$, as reflected by the absence of off-diagonal elements in ${\left(\varepsilon_{xyz,p-Si}\right)}_{matrix}$ (Eq. (17)). For 〈111〉-Si-wafer, although all the shear components of $\xi_{123,Si}$ are nonzero after the conversion by $T_{\xi }$, they are identical in magnitude and 120° difference in phase with respect to *z*-axis one to each other in G-CSYS. Thus, the mutual neutralization of these shear components leads to absence of off-diagonal elements in ${\left(\varepsilon_{xyz,p-Si}\right)}_{matrix}$. As the off-diagonal elements in ${\left(\varepsilon_{xyz,p-Si}\right)}_{matrix}$ depict the coupling effect between different components of the electric field, absence of them indicates there is no shift of the electric energy from *s*- to *p*-states of polarization, and therefore the *p*-to-*s* intensity ratio remains constantly zero for the case of 〈100〉- and 〈111〉-Si-wafers (Fig. 9 (a) and (b)).

In contrast to the 〈100〉- and 〈111〉-Si-wafers, clear *p*-to-*s* intensity ratios are observed in 〈110〉-Si-wafers, owing to the presence of a nonzero off-diagonal element in its ${\left(\varepsilon_{xyz,p-Si}\right)}_{matrix}$, *i.e.*, $\varepsilon_{6,xyz,p-Si}$. The maximum and minimum *p*-to-*s* intensity ratios are observed, respectively, when $\theta_{wafer}=45^{{}^{\circ}}$ ($135^{{}^{\circ}}$, $225^{{}^{\circ}}$, or $315^{{}^{\circ}}$) and $0^{{}^{\circ}}$ ($90^{{}^{\circ}}$, $180^{{}^{\circ}}$, or $270^{{}^{\circ}}$) which are in line with those of $\left|\varepsilon_{6,xyz,p-Si}\right|$ (Fig. 9 (c)).

The experimental results quantitatively corroborate the theoretical prediction of the *p*-to-*s* intensity ratios that are obtained using (13), (14), (15), (16), (17), (18), (19), (20), (21), (22), (23), (24), (25), (26), (27), (28), (29), (30), (31), (32), (33), (34), (35), (36), for all three types of crystal orientations of Si-wafers.

### 5.2 Azimuth and ellipticity angle

**Fig. 10:**
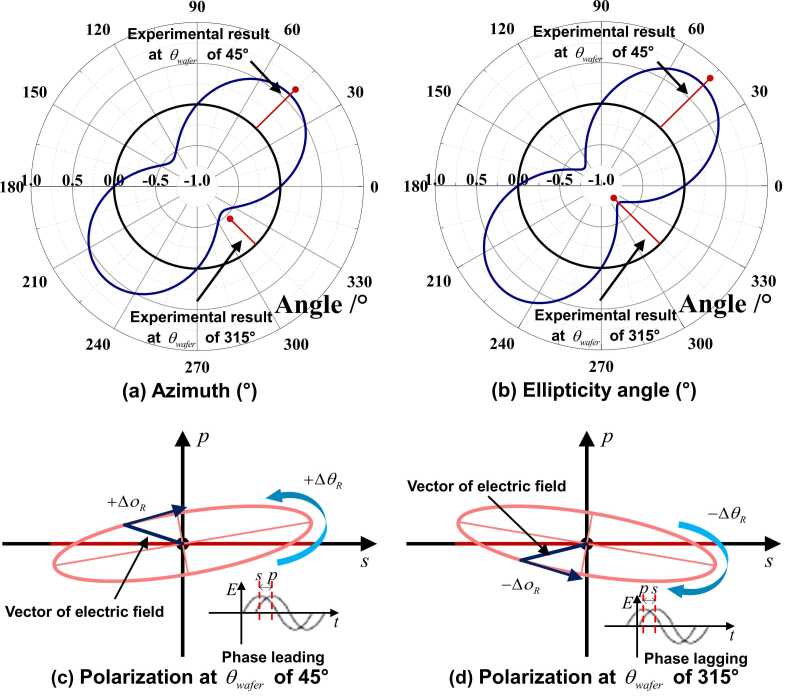
Calculated variation (blue curves) of (a) azimuth and (b) ellipticity angle corresponding to $\theta_{wafer}$ in 〈110〉-Si-wafer (in (a) and (b), experimental data exhibited using the red points linked with the 0-baseline by red lines, with respect to $\theta_{wafer}$ of 45° and 315°, are also included, respectively), and the polarization at $\theta_{wafer}$ of (c) 45° and (d) 315° (the proportion between the length of minor and major axes is exaggerated to highlight the changed polarization).

As derived by Eq. (37), the non-zero *p*-state of polarization is associated with both the azimuth and ellipticity angle of the polarization ellipse. This observation quantitatively determines the rotation of the major axis of polarization, and the magnitude of the minor axis, respectively. Fig. 10 (a) and (b) present the theoretical results of the azimuth and ellipticity angle along with $\theta_{wafer}$. Comparing the theoretical results in Fig. 10 (a), (b), and 9 (c), it is apparent that variation of $\theta_{wafer}$, corresponding to the maximum and minimum absolute values of the azimuth and ellipticity angle, is in accordance with that of the *p*-to-*s* intensity ratio. In addition, for $\theta_{wafer}$ of 45° and 225°, azimuth and ellipticity angle positively shift, while negatively shift when $\theta_{wafer}$= 135° and 315°. The positively or negatively shifted azimuth is attributed to either the anticlockwise or clockwise rotation of the major axis of the polarization, respectively, as shown in Fig. 10 (c) and (d). Representative results from experiment, showing the variation of azimuth and ellipticity angle when $\theta_{wafer}$ is 45° or 315°, are displayed in Fig. 10 (a) and (b). The experimental observation qualitatively affirms the same positive or negative trend of the variation of azimuth and ellipticity angle as shown in theoretical results calculated using Eq. (37) and figuratively displayed in Fig. 10 (c) and (d).

For ellipticity angle, irrespective of positive or negative shift, it does not directly change the shape of the ellipse representing the polarization (Fig. 10 (c) and (d)). Instead, it indicates that the vector of the electric field of the reflected probe beam rotates either clockwise or anticlockwise with regard to its propagation direction. In addition, the shift of the ellipticity angle can also be attributed to the shift of the phase of the *p*-component of the electric field advances or lags compared to that of the *s*-component.

### 5.3 Selective observation of shear strains

**Fig. 11:**
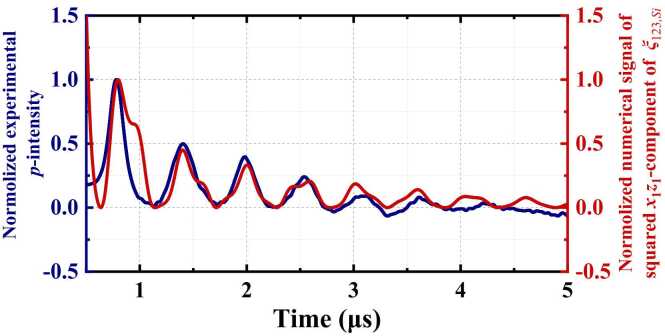
Temporal *p*-intensity signal acquired in the 〈110〉-Si-wafer at the $\theta_{wafer}$ of 45° (blue), and the corresponding numerical signal of squared *x*_1_*z*_1_-component of $\xi_{123,Si}$ (red).

Ascertainment of the relationship between the strain and the polarization of the probe beam facilitates selective acquisition of shear strains according to the perturbation of shear strains to the polarization. Fig. 11 compares the experimentally obtained temporal *p*-intensity signal in the 〈110〉-Si-wafer with respect to the $\theta_{wafer}$ of 45° using set-up-2, and the corresponding numerical results of the squared *x*_1_*z*_1_-component of $\xi_{123,Si}$. Here, selecting the *x*_1_*z*_1_-component of $\xi_{123,Si}$ for the comparison is because it is the only non-zero shear component existing in a 〈110〉-Si-wafer based on Eq. (12). As the original *p*-intensity signal is zero, only the positive shift of the *p*-intensity is observed. Recalling the theoretical illustration in Section 3 and the quadratic relationship between the intensity and the electric field, it is noted that the intensity is proportional to the square of shear strains, instead of the shear strains *per se*. These can be affirmed by the quantitative match between the experimental and calculated signals in Fig. 11. Previous results of the *p*-to-*s* intensity ratio have affirmed non-perturbation to the polarization when shear strains are absent. Here the quantitative match between the experimentally measured and calculated signals in Fig. 11 corroborates that the developed OA approach is capable of selectively measuring dynamic signals of the shear strains. In addition, the quantitative match in the waveform also demonstrates a reliable estimation of the elastic properties of the anisotropic MS.

## 6 Concluding remarks

For interrogating the shear strains induced by ultrasonic bulk waves in opaque, anisotropic MS in a contactless and selective manner, an OA approach is developed, in conjunction with the multiphysics modeling. The multiphysics model theoretically interprets the coupling between shear strains and the polarization of the probe beam, which is governed by multiple factors including the mechanical, photoelastic, and strain-induced optical anisotropies of MS. The accuracy of the developed OA approach for selective observation of shear-strains in MS-wafers is verified experimentally, in which the shear-strain-induced perturbation to the optical polarization and the corresponding temporal signals are measured in Si-wafers of different crystal orientations. The results confirm the presence of distinct crystal-orientation-dependence and crystal-structure-related symmetry of the perturbation to the optical polarization. In addition, the existence of shear strains and their perturbation to the polarization of the reflected probe beam are associated with the crystal orientations, suggesting the shear-strain sensitivity of the developed OA approach in noncontact characterization and inspection of anisotropic MS. This selective sensitivity is further affirmed by the match between experimental *p*-intensity and numerical squared shear-strain signals. For an MS wafer with known crystal orientation, the developed approach can selectively acquire the shear strains of OA-UBW with ultralow magnitude of microscale. Combined with the fully noncontact implementation and the selective sensitivity of shear strains, the presented OA method exhibits transdisciplinary application prospects, for instance the *in situ* characterization of the microscale anisotropic properties of monocrystalline silicon wafers in electronics industry.

## CRediT authorship contribution statement

All the authors have jointly made contribution to this paper, including: **Yi He:** Conceptualization, Methodology, Experiment, Validation, Writing – original draft. **Hoon Sohn:** Methodology, Investigation, Writing – review & editing. **Osamu Matsuda:** Methodology, Investigation, Writing – review & editing. **Zhongqing Su:** Conceptualization, Methodology, Supervision, Funding acquisition, Writing – review & editing.

## Declaration of Competing Interest

The authors declare the following financial interests/personal relationships which may be considered as potential competing interests: Zhongqing Su reports financial support was provided by Hong Kong Research Grants Council. Hoon Sohn reports financial support was provided by Korean government (MSIT). Osamu Matsuda reports financial support was provided by Japan Society for the Promotion of Science.

## Data Availability

Data will be made available on request.

## References

[1] Mack C.A. (2011). Fifty years of Moore's law. IEEE Trans. Semicond. Manuf..

[2] Theis T.N., Wong H.-S.P. (2017). The end of moore's law: A new beginning for information technology. Comput. Sci. Eng..

[3] Zhang Z., Yan J., Kuriyagawa T. (2019). Manufacturing technologies toward extreme precision. Int. J. Extrem. Manuf..

[4] del Barrio J., Sánchez‐Somolinos C. (2019). Light to shape the future: from photolithography to 4D printing. Adv. Opt. Mater..

[5] Choudhury, D., 2010, 3D integration technologies for emerging microsystems. 2010 IEEE MTT-S international microwave symposium, Anaheim, CA, USA. https://doi.org/10.1109/MWSYM.2010.5514747.

[6] Shulaker M.M., Hills G., Park R.S., Howe R.T., Saraswat K., Wong H.-S.P., Mitra S. (2017). Three-dimensional integration of nanotechnologies for computing and data storage on a single chip. Nature.

[7] Lau J.H. (2014). Overview and outlook of three-dimensional integrated circuit packaging, three-dimensional Si integration, and three-dimensional integrated circuit integration. J. Electron. Packag..

[8] Wang Z.L., Wu W., Falconi C. (2018). Piezotronics and piezo-phototronics with third-generation semiconductors. MRS Bull..

[9] Schönfelder G., Liess M. (2022). Sensors in Science and Technology: Functionality and Application Areas.

[10] Van Zant P. (2014). https://www.accessengineeringlibrary.com/content/book/9780071821018.

[11] Liu C. (2012). https://compress-pdf.obar.info/download/compresspdf.

[12] Khan A.I., Keshavarzi A., Datta S. (2020). The future of ferroelectric field-effect transistor technology. Nat. Electron..

[13] Tu K.-N. (2011). Reliability challenges in 3D IC packaging technology. Microelectron. Reliab..

[14] Sukumaran V., Kumar G., Ramachandran K., Suzuki Y., Demir K., Sato Y., Tummala R.R. (2014). Design, fabrication, and characterization of ultrathin 3-D glass interposers with through-package-vias at same pitch as TSVs in silicon. IEEE Trans. Compon., Packag. Manuf. Technol..

[15] Aryan P., Sampath S., Sohn H. (2018). An overview of non-destructive testing methods for integrated circuit packaging inspection. Sensors.

[16] Su L., Wang L., Li K., Wu J., Liao G., Shi T., Lin T. (2019). Automated X-ray recognition of solder bump defects based on ensemble-ELM. Sci. China Technol. Sci..

[17] Yang J., Choi J., Hwang S., An Y.-K., Sohn H. (2016). A reference-free micro defect visualization using pulse laser scanning thermography and image processing. Meas. Sci. Technol..

[18] Nakamae K. (2021). Electron microscopy in semiconductor inspection. Meas. Sci. Technol..

[19] Kitami, K., Takada, M., Kikuchi, O., & Ohno, S. (2013). Development of high resolution scanning aeoustie tomograph for advanced LSI packages. Proceedings of the 20th IEEE International Symposium on the Physical and Failure Analysis of Integrated Circuits (IPFA), Suzhou. https://doi.org/10.1109/IPFA.2013.6599215.

[20] Verrina, V., 2021, Laser-induced ultrasound for the detection of buried micro-and nano-structures [Doctoral thesis, University of Amsterdam]. Amsterdam. 〈https://ir.arcnl.nl/pub/164/2021-Vanessa-Verrina.pdf〉.

[21] Zhang H., Antoncecchi A., Edward S., Planken P., Witte S. (2021). Ultrafast laser-induced guided elastic waves in a freestanding aluminum membrane. Phys. Rev. B.

[22] Liu P., Yi K., Sohn H. (2021). Estimation of silicon wafer coating thickness using ultrasound generated by femtosecond laser. J. Nondestruct. Eval., Diagn. Progn. Eng. Syst..

[23] Matsuda O., Larciprete M.C., Voti R.L., Wright O.B. (2015). Fundamentals of picosecond laser ultrasonics. Ultrasonics.

[24] Wissmeyer G., Pleitez M.A., Rosenthal A., Ntziachristos V. (2018). Looking at sound: optoacoustics with all-optical ultrasound detection. Light.: Sci. Appl..

[25] Zhang L., Cai Y., Li L., Feng W., Wen R.-T., Shin S., Guo L. (2023). Metal transducer-assisted acoustic deformation potential characterization via coherent acoustic phonon dynamics. Photoacoustics.

[26] He Y., Wang K., Xu L., Sohn H., Su Z. (2023). Laser ultrasonic imaging of submillimeter defect in a thick waveguide using entropy-polarized bilateral filtering and minimum variance beamforming. Mech. Syst. Signal Process..

[27] Kim T., Chang W.-Y., Kim H., Jiang X. (2019). Narrow band photoacoustic lamb wave generation for nondestructive testing using candle soot nanoparticle patches. Appl. Phys. Lett..

[28] Ruello P., Gusev V.E. (2015). Physical mechanisms of coherent acoustic phonons generation by ultrafast laser action. Ultrasonics.

[29] Zheng C., Zhu H., Xu Z., Sinha R.K., Li Q., Ghosh P. (2021). High-efficient photoacoustic generation with an ultrathin metallic multilayer broadband absorber. Opt. Express.

[30] Ritzmann U., Oppeneer P.M., Maldonado P. (2020). Theory of out-of-equilibrium electron and phonon dynamics in metals after femtosecond laser excitation. Phys. Rev. B.

[31] Dehoux T., Perton M., Chigarev N., Rossignol C., Rampnoux J.-M., Audoin B. (2006). Effect of laser pulse duration in picosecond ultrasonics. J. Appl. Phys..

[32] Anisimov S., Kapeliovich B., Perelman T. (1974). Electron emission from metal surfaces exposed to ultrashort laser pulses. Zh. Eksp. Teor. Fiz..

[33] Thomsen C., Grahn H.T., Maris H.J., Tauc J. (1986). Surface generation and detection of phonons by picosecond light pulses. Phys. Rev. B.

[34] Tas G., Maris H.J. (1994). Electron diffusion in metals studied by picosecond ultrasonics. Phys. Rev. B.

[35] Liu P., Sohn H. (2016). Numerical simulation of damage detection using laser-generated ultrasound. Ultrasonics.

[36] Thompson D., Gasteau D., Manohar S. (2020). Spatially compounded plane wave imaging using a laser-induced ultrasound source. Photoacoustics.

[37] Zhang H., Antoncecchi A., Edward S., Setija I., Planken P., Witte S. (2020). Unraveling phononic, optoacoustic, and mechanical properties of metals with light-driven hypersound. Phys. Rev. Appl..

[38] Białek R., Vasileiadis T., Pochylski M., Graczykowski B. (2023). Fano meets Stokes: Four-order-of-magnitude enhancement of asymmetric Brillouin light scattering spectra. Photoacoustics.

[39] Xie Q., Mezil S., Otsuka P.H., Tomoda M., Laurent J., Matsuda O., Wright O.B. (2019). Imaging gigahertz zero-group-velocity Lamb waves. Nat. Commun..

[40] Wright C., Hartland G.V. (2023). Mode specific dynamics for the acoustic vibrations of a gold nanoplate. Photoacoustics.

[41] Maris, H.J., Antonelli, G.A., Ford, W.K., Morath, C.J., Stoner, R.J., & Tas, G., 2006, Non‐Destructive Testing Using Picosecond Ultrasonics. AIP Conference Proceedings, Brunswick, Maine. https://doi.org/10.1063/1.2184531.

[42] Hajireza P., Shi W., Bell K., Paproski R.J., Zemp R. (2017). Non-interferometric photoacoustic remote sensing microscopy. Light.: Sci. Appl..

[43] Jang J., Liu P., Kim B., Kim S.-w, Sohn H. (2020). Silicon wafer crack detection using nonlinear ultrasonic modulation induced by high repetition rate pulse laser. Opt. Lasers Eng..

[44] Flizikowski G., Capeloto O., Camargo V., Anghinoni B., Baesso M.L., Malacarne L.C., Astrath N.G.C. (2020). Laser induced thermoelastic surface displacement in solids detected simultaneously by photothermal mirror and interferometry. Opt. Express.

[45] Pupeikis J., Willenberg B., Bruno F., Hettich M., Nussbaum-Lapping A., Golling M., Camy P. (2021). Picosecond ultrasonics with a free-running dual-comb laser. Opt. Express.

[46] Yi K., Liu P., Park S.-H., Sohn H. (2022). Femtosecond laser ultrasonic inspection of a moving object and its application to estimation of silicon wafer coating thickness. Opt. Lasers Eng..

[47] Liu P., Yi K., Park Y., Sohn H. (2022). Ultrafast nonlinear ultrasonic measurement using femtosecond laser and modified lock-in detection. Opt. Lasers Eng..

[48] Antoncecchi A., Zhang H., Edward S., Verrina V., Planken P.C., Witte S. (2020). High-resolution microscopy through optically opaque media using ultrafast photoacoustics. Opt. Express.

[49] Pezeril T. (2016). Laser generation and detection of ultrafast shear acoustic waves in solids and liquids. Opt. Laser Technol..

[50] Decremps F., Belliard L., Gauthier M., Perrin B. (2010). Equation of state, stability, anisotropy and nonlinear elasticity of diamond-cubic (ZB) silicon by phonon imaging at high pressure. Phys. Rev. B.

[51] Wang Y., Khafizov M. (2021). Shear wave generation by mode conversion in picosecond ultrasonics: Impact of grain orientation and material properties. J. Am. Ceram. Soc..

[52] Thomas S., Manju M., Ajith K., Lee S., Zaeem M.A. (2020). Strain-induced work function in h-BN and BCN monolayers. Phys. E: Low. -Dimens. Syst. Nanostruct..

[53] Gusev V. (2009). On generation of picosecond inhomogeneous shear strain fronts by laser-induced gratings. Appl. Phys. Lett..

[54] Matsuda O., Wright O., Hurley D., Gusev V., Shimizu K. (2008). Coherent shear phonon generation and detection with picosecond laser acoustics. Phys. Rev. B.

[55] Matsuda O., Wright O., Hurley D., Gusev V., Shimizu K. (2004). Coherent shear phonon generation and detection with ultrashort optical pulses. Phys. Rev. Lett..

[56] Kouyaté M., Pezeril T., Gusev V., Matsuda O. (2016). Theory for optical detection of picosecond shear acoustic gratings. JOSA B.

[57] Matsuda O., Tsutsui K., Vaudel G., Pezeril T., Fujita K., Gusev V. (2020). Optical generation and detection of gigahertz shear acoustic waves in solids assisted by a metallic diffraction grating. Phys. Rev. B.

[58] Pezeril T., Ruello P., Gougeon S., Chigarev N., Mounier D., Breteau J.-M., Gusev V. (2007). Generation and detection of plane coherent shear picosecond acoustic pulses by lasers: Experiment and theory. Phys. Rev. B.

[59] Saito T., Matsuda O., Tomoda M., Wright O.B. (2010). Imaging gigahertz surface acoustic waves through the photoelastic effect. JOSA B.

[60] Stoehr M., Gerlach G., Härtling T., Schoenfelder S. (2020). Analysis of photoelastic properties of monocrystalline silicon. J. Sens. Sens. Syst..

[61] Herms M., Irmer G., Kupka G., Kirchner N., Wagner M. (2020). Comparative Study of the Photoelastic Anisotropy of Si and GaAs. J. Electron. Mater..

[62] Haynes W.M. (2014).

[63] Werner W.S., Glantschnig K., Ambrosch-Draxl C. (2009). Optical constants and inelastic electron-scattering data for 17 elemental metals. J. Phys. Chem. Ref. Data.

[64] Hopcroft M.A., Nix W.D., Kenny T.W. (2010). What is the Young's Modulus of Silicon. J. Micro Syst..

[65] Vogt, M.R., 2016, Development of physical models for the simulation of optical properties of solar cell modules [Doctoral thesis, University of Hannover]. Hannover. 〈https://www.researchgate.net/profile/Malte-Ruben-Vogt-2/publication/303300115_Development_of_Physical_Models_for_the_Simulation_of_Optical_Properties_of_Solar_Cell_Modules/links/573b9ff308aea45ee840670a/Development-of-Physical-Models-for-the-Simulation-of-Optical-Properties-of-Solar-Cell-Modules.pdf〉.

[66] Ciddor P.E. (1996). Refractive index of air: new equations for the visible and near infrared. Appl. Opt..

[67] Ledbetter H. (1977). Elastic properties of zinc: A compilation and a review. J. Phys. Chem. Ref. Data.

